# Supplementary material for: Ecology and spread of the North American H5N1 epizootic
Source: Nature. 2025 Nov 12;649(8096):432–41. doi: 10.1038/s41586-025-09737-x (PMC12779553; doi:10.1038/s41586-025-09737-x)
Supplement: Supplementary file 1 — Supplementary Figs. 1–26 and Supplementary Tables 1–18. [file 41586_2025_9737_MOESM1_ESM.pdf]

nature portfolio

https://doi.org/10.1038/s41586-025-09737-x

Supplementary information

Ecology and spread of the North American H5N1 epizootic

In the format provided by the authors and unedited

Supplemental Material: Ecology and spread of the North American H5N1 epizootic

Lambodhar Damodaran[1], Anna Jaeger[1], Louise H. Moncla[1]

[1]Department of Pathobiology, School of Veterinary Medicine, University of Pennsylvania

Corresponding Author: Louise H. Moncla
Email: lhmoncla@upenn.edu

Contents

Supplementary Figures and Tables are provided here in the following order

Supplementary Figures:

Sup. Figure 1. Detections of HPAI by sampling method and species.
Sup. Figure 2. Number of HA isolates for HPAI in North America in GISAID
Sup. Figure 3. Effective population size estimates for host orders analyses
Sup. Figure 4. Correlation between effective population size and detections of HPAI
Sup. Figure 5. Cross correlation plots between effective population size and detections of HPAI
Sup. Figure 6. Phylogeny for analyses of host migratory behavior.
Sup. Figure 7. MCC trees and bar plots for transition rates for each host order subsample analysis.
Sup. Figure 8. Combined bar plots for transition rates for equal and proportional sampling methods combined.
Sup. Figure 9. Number of transitions between host orders across MCC tree.
Sup. Figure 10. Exploded tree views of MCC for each host order transition.
Sup. Figure 11. Two state rarefaction analyses MCC trees
Sup. Figure 12. Exploded tree views of two state rarefaction MCC trees (wild origin).
Sup. Figure 13. Exploded tree views of two state rarefaction MCC trees (domestic origin).
Sup. Figure 14. MCC and exploded tree views for analyses of domestic:wild analyses with turkey sequences.
Sup. Figure 15. Exploded tree views of three state titration analyses.
Sup. Figure 16. Number of sequences and detections for backyard birds and commercial birds over time.
Sup. Figure 17. Number of sequences over time by domestic status
Sup. Figure 18. Number of sequences for each taxonomic order available.
Sup. Figure 19. Root probability for global geographic DTA (Discrete trait Analyses) shuffle test.
Sup. Figure 20. Root probability for migration DTA shuffle test.
Sup. Figure 21. Root probability for USFWS flyway DTA shuffle test.

Sup. Figure 22. Root probability for host order (equal) DTA shuffle test.
Sup. Figure 23. Root probability for host order (proportional) DTA shuffle test.
Sup. Figure 24. Root probability for domestic:wild DTA shuffle test.
Sup. Figure 25. Root probability for domestic:wild turkey DTA shuffle test.
Sup. Figure 26. Root probability for backyard bird DTA shuffle test.

Supplementary Tables:

Sup. Table 1. DTA results for USFWS flyways.
Sup. Table 2. DTA results for geographic group based on latitude.
Sup. Table 3. DTA results for migratory behavior.
Sup. Table 4. DTA results for host order (equal) combined results.
Sup. Table 5. DTA results for host order (proportional) combined results.
Sup. Table 6. DTA results for host order (equal subsample 1).
Sup. Table 7. DTA results for (equal subsample 2).
Sup. Table 8. DTA results for (equal subsample 3).
Sup. Table 9. DTA results for (proportional subsample 1).
Sup. Table 10. DTA results for (proportional subsample 2).
Sup. Table 11. DTA results for (proportional subsample 3).
Sup. Table 12. Transitions to domestic and wild in each rarefaction analyses.
Sup. Table 13. Number of transitions for each state pair in three state rarefaction.
Sup. Table 14. DTA results for titration analyses (25% wild birds).
Sup. Table 15. DTA results for titration analyses (50% wild birds).
Sup. Table 16. DTA results for titration analyses (75% wild birds).
Sup. Table 17. DTA results for titration analyses (100% wild birds).
Sup. Table 18. GISAID acknowledgements table.

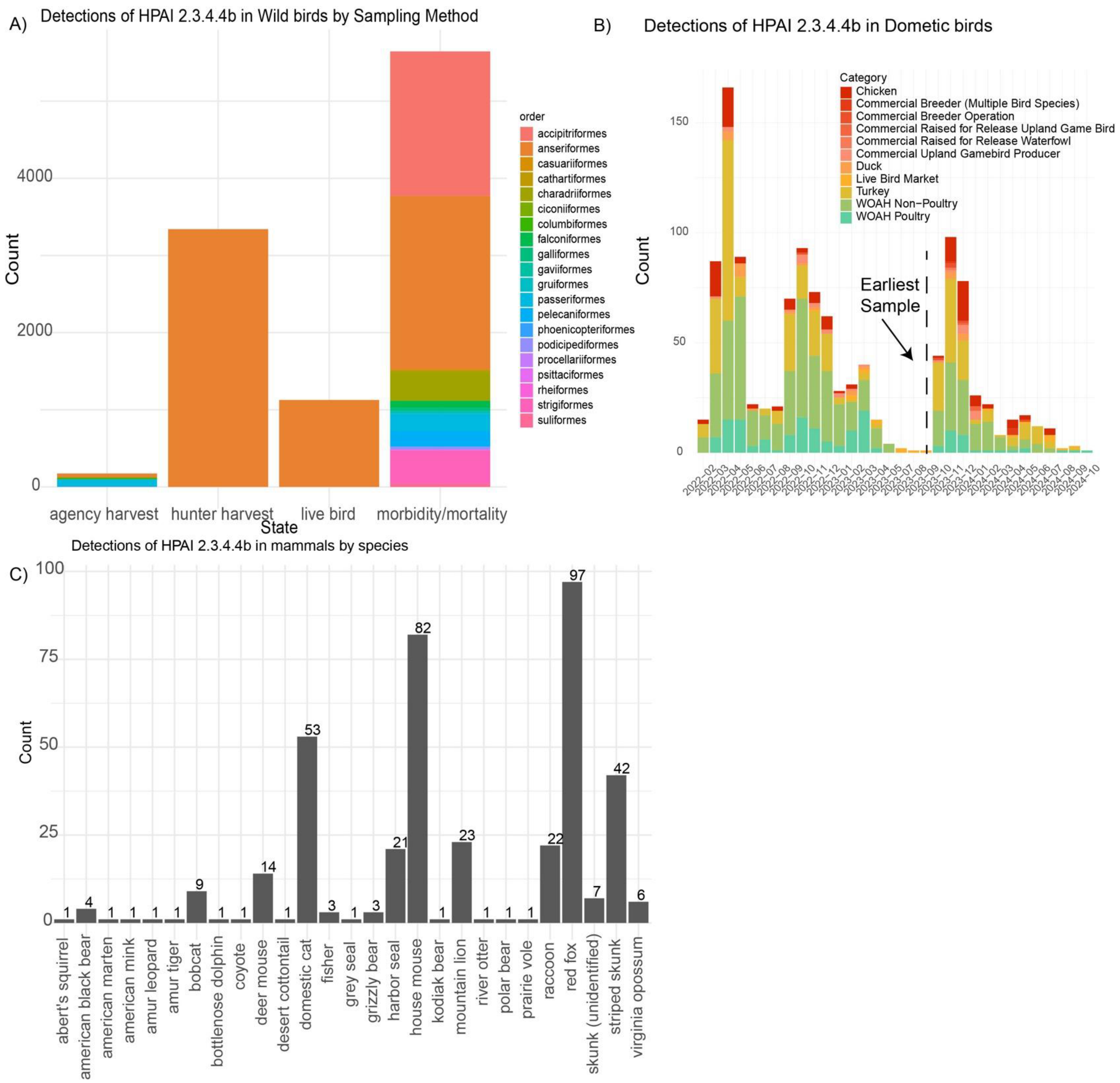

Figure S1. A) Number of detections of HPAI in North American wild birds by collection method. Morbidity and mortality refer to sick or dead birds. B) Domestic bird detections of HPAI in North America on a monthly basis colored by production type. C) Number of detections in mammals by species.

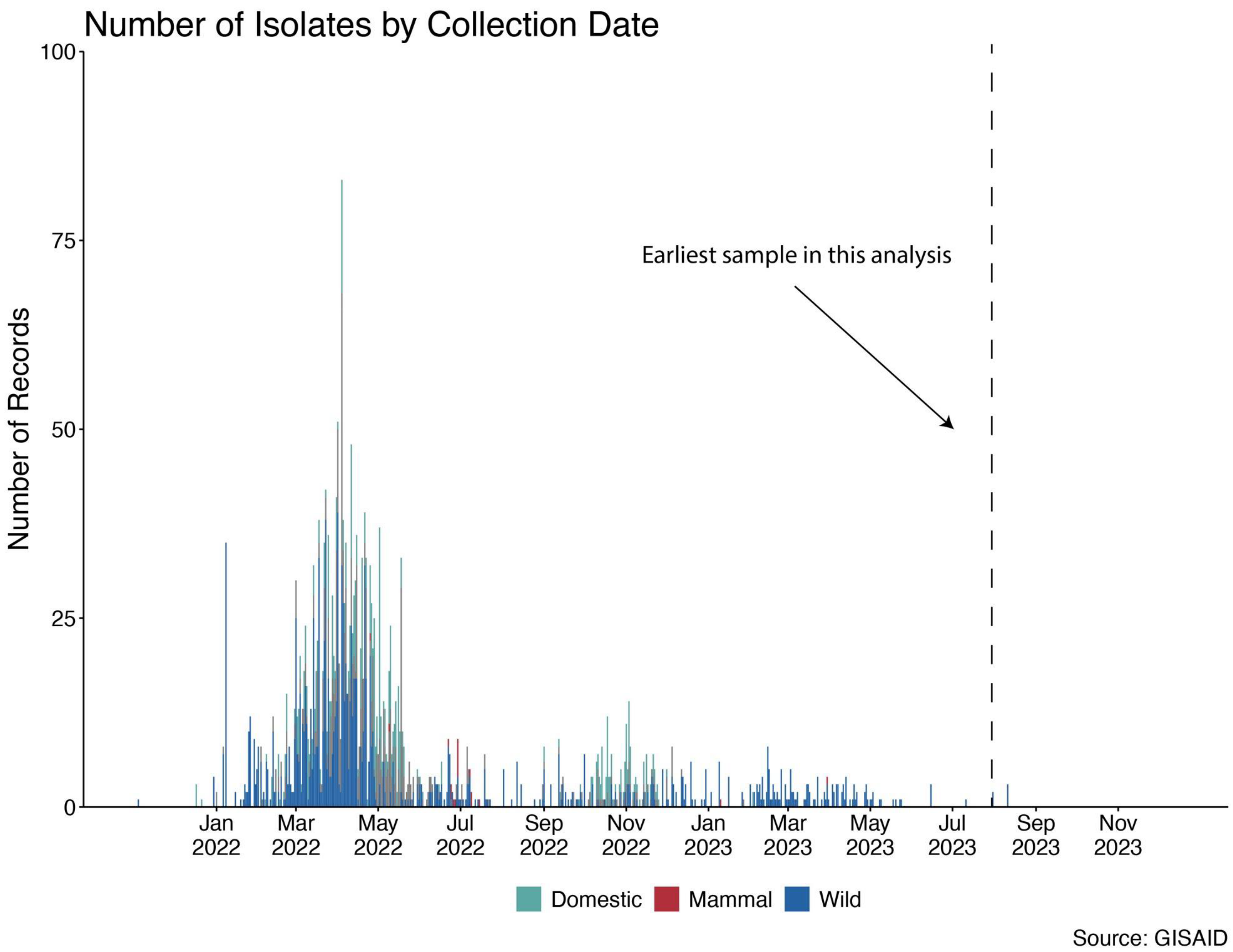

Figure S2. Number of HA isolates for HPAI in North America by collection date available in GISAID.

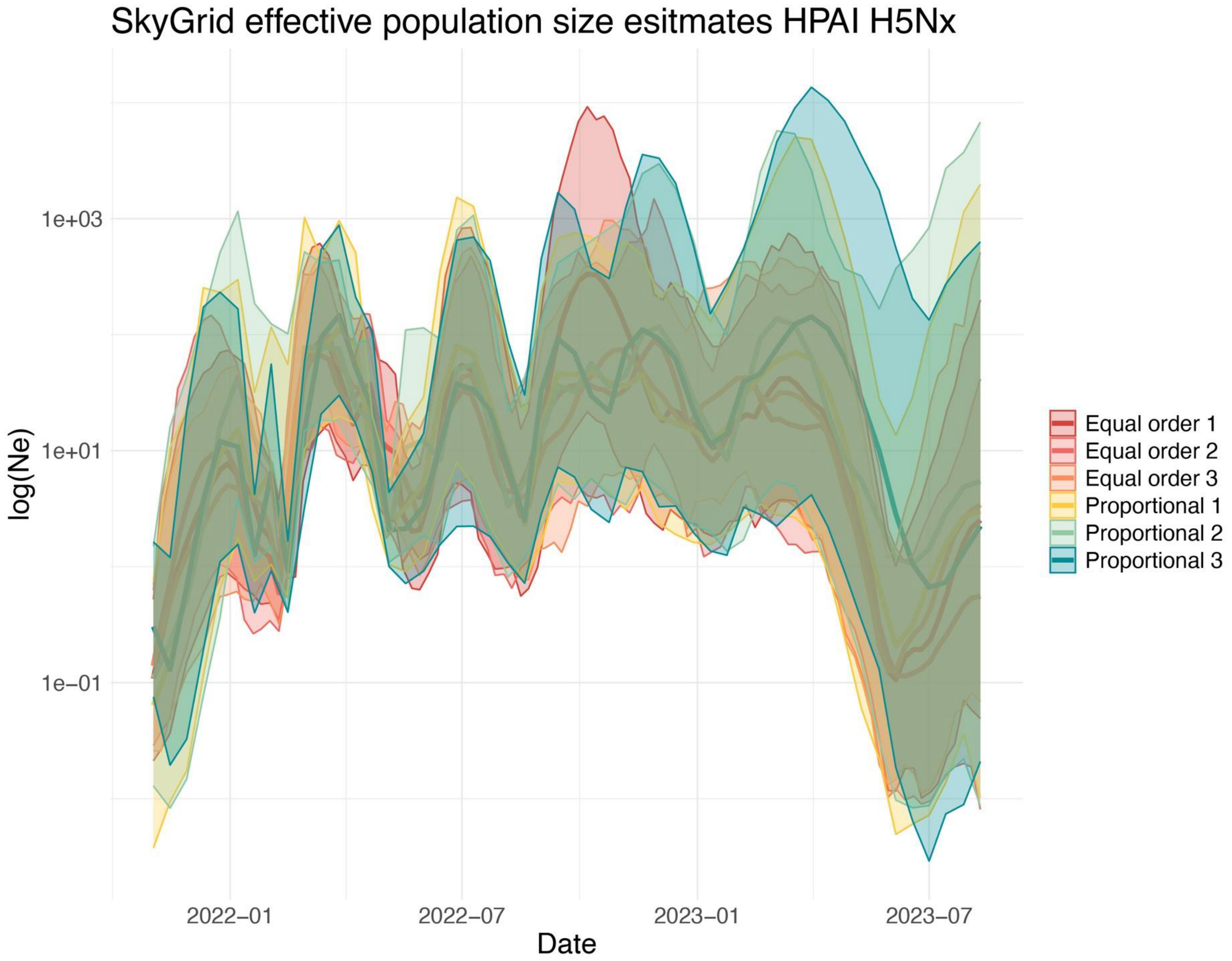

Figure S3. A) SkyGrid Coalescent effective population size reconstructions for six different subsamples of HPAI in North America between 2021 and 2023. Three subsamples with equal proportions of host orders and three subsamples with number of sequences proportional to detections in those hosts.

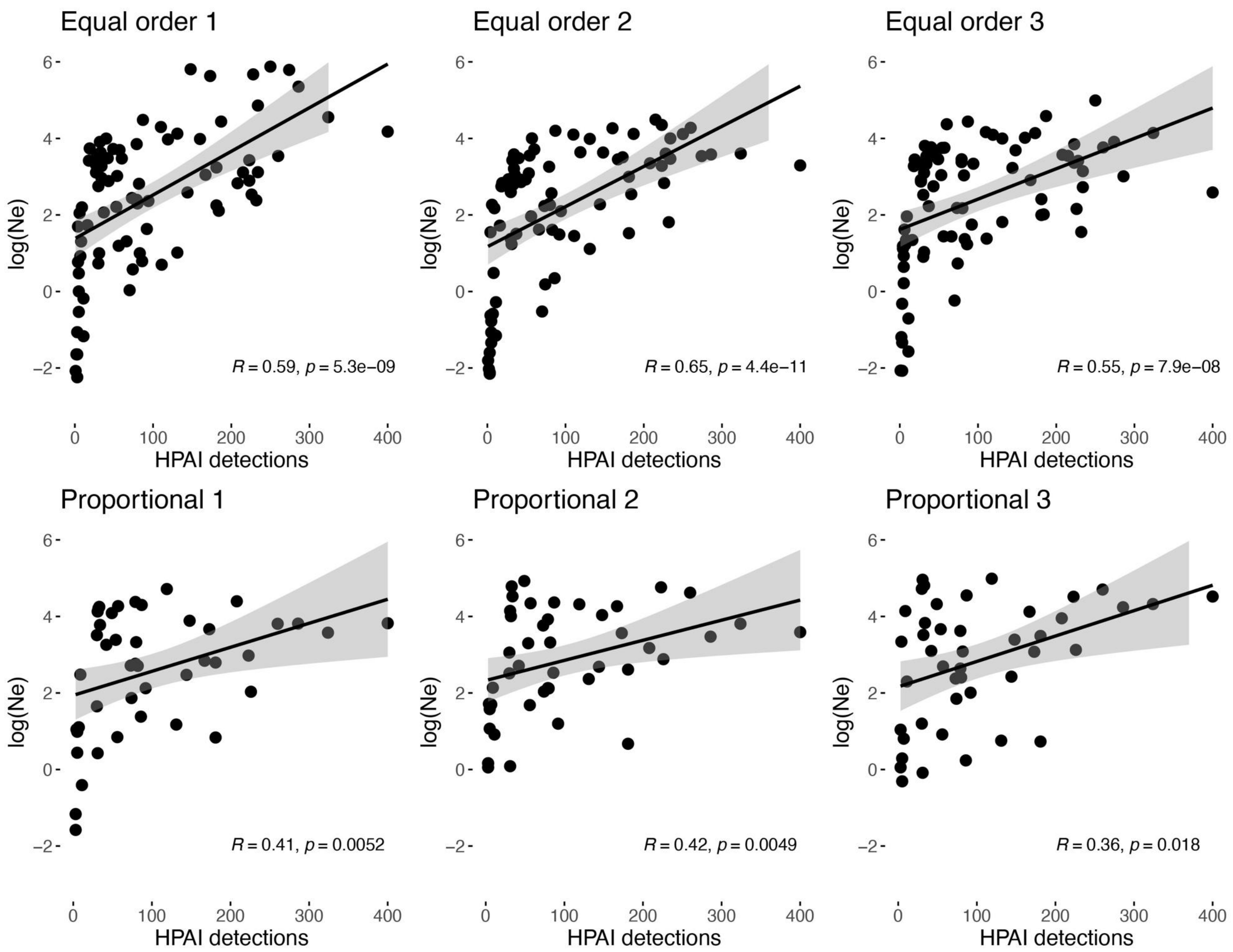

Figure S4. Spearman correlation plot of effective population size estimates vs detections at corresponding timepoints.

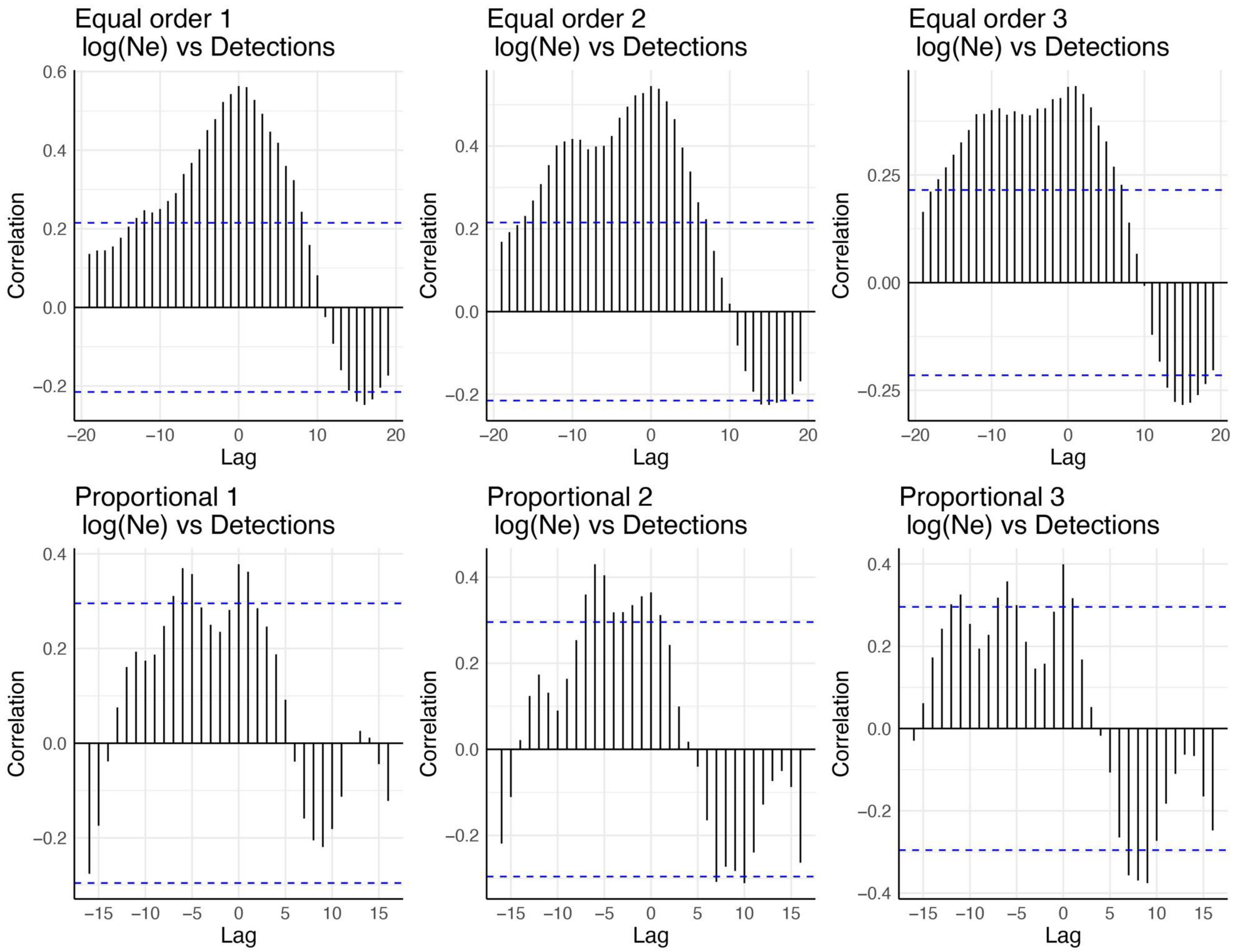

Figure S5. Cross correlation plot of effective population size estimates and detections of HPAI. The x-axis represents the time lag in weeks and the y-axis represents the correlation. Dotted lines represent the significance thresholds.

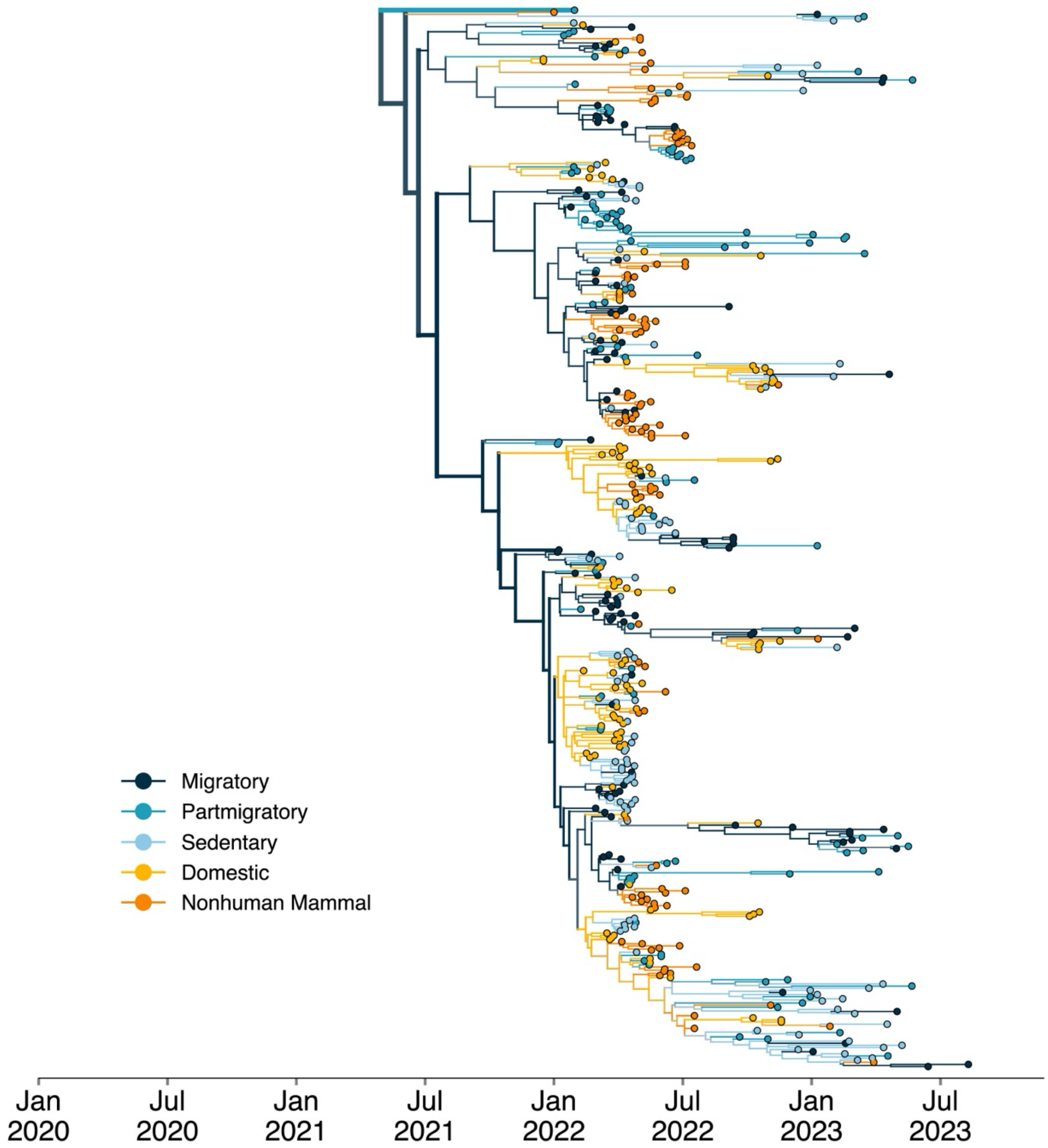

Figure S6. Phylogenetic reconstruction of n=1000 sequences colored by Migratory behavior of host (Determined using AVONET). Color corresponds to the migratory behavior of the host inferred for the branches and for the host of the tip.

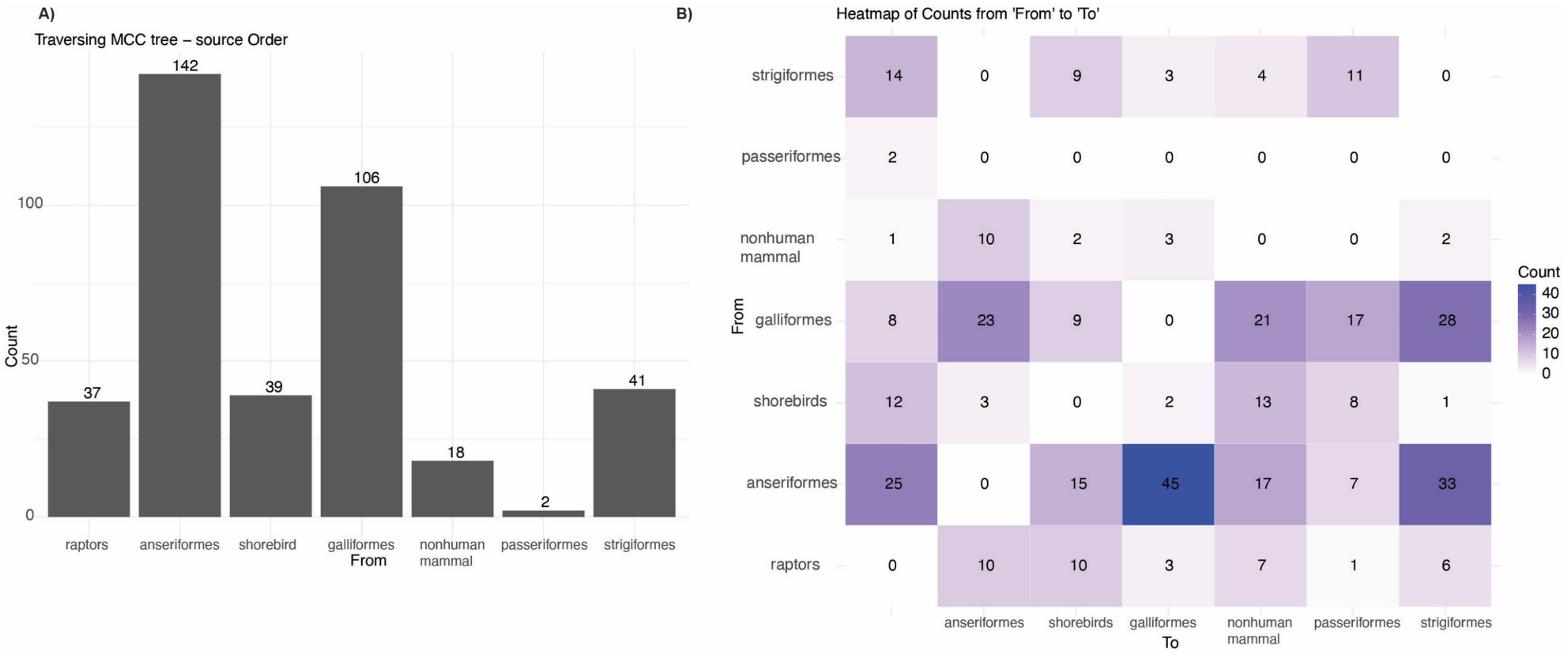

Figure S7. Results from traversing MCC tree and enumerating number of transitions between host orders. A) Number of times a given host order transition to another host order. B) Number of transitions between each host order pair. Transitions calculated using the BALTIC python package.

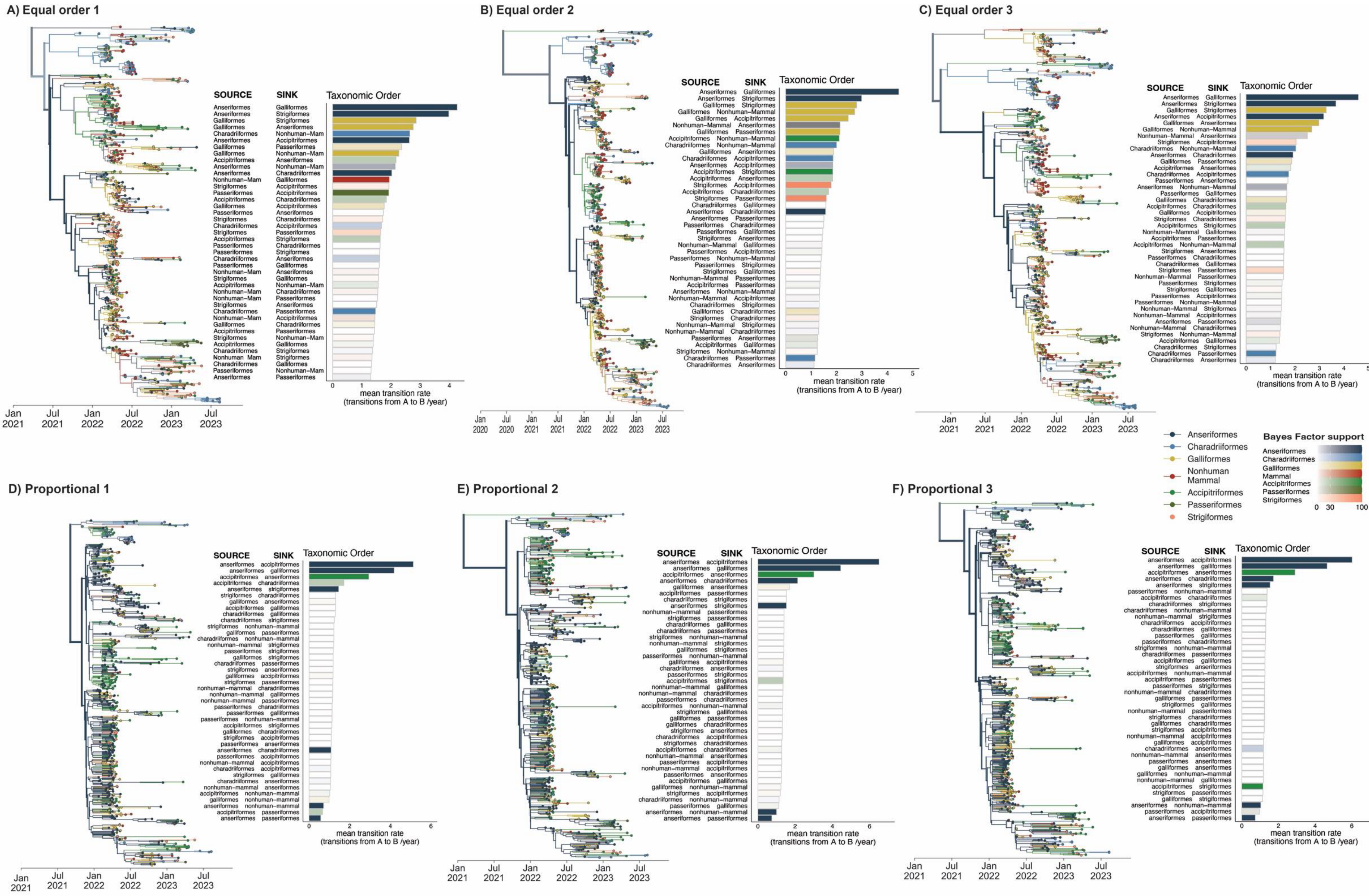

Figure S8: MCC trees for each subsampled dataset for analysis of host order transmission where colors correspond to host Order. with corresponding bar plot showing discrete trait rates. A-C) are equal number of order datasets and D-F) are case proportional datasets.

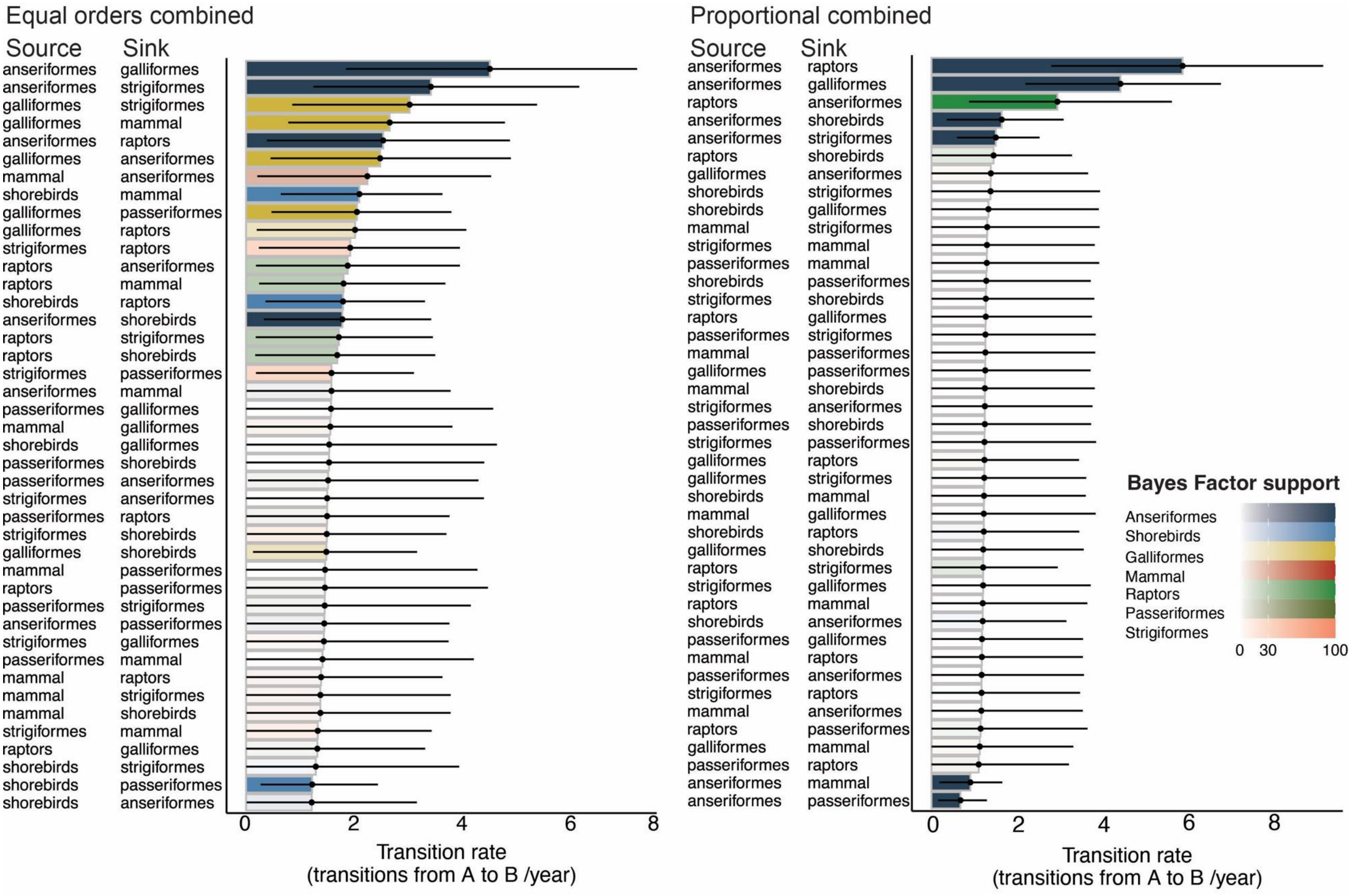

Figure S9. Transition rates (dot represents mean transition and bars represent 95% HPD) from combined results of the BSSVS of combined results for equal orders schema (Left) and proportional scheme (left) where color of the bar corresponds to the source population and the opacity corresponds to the bayes factor support (where white corresponds to BF < 3 and full color corresponds to BF > 100).

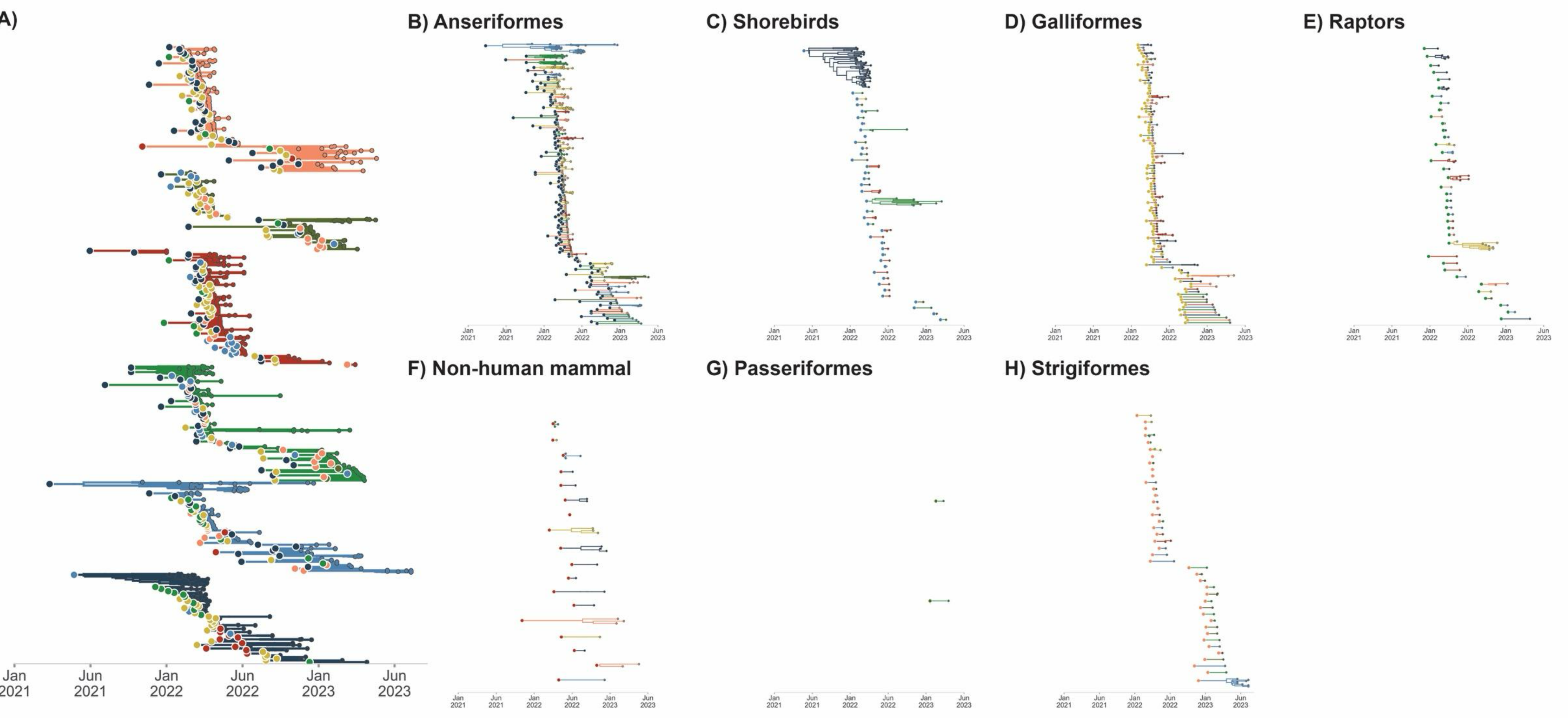

Figure S10: A) Exploded tree view of the MCC tree of equal order phylogenetic reconstruction where subtrees represent the traversal of a tree from the root to the tip where the state is unchanged from the initial state (given by the large dot on left) to the tips represented by the smaller dots representing continuous chains of transmission within a given state. Colors correspond to the state of a given tip, branch node. B-H represent the exploded trees faceted by taxonomic host at the origin: B) Anseriformes, C) Shorebirds , D) Galliformes, E) Raptors, F) Non-human mammal, G) Passeriformes, H) Strigiformes.

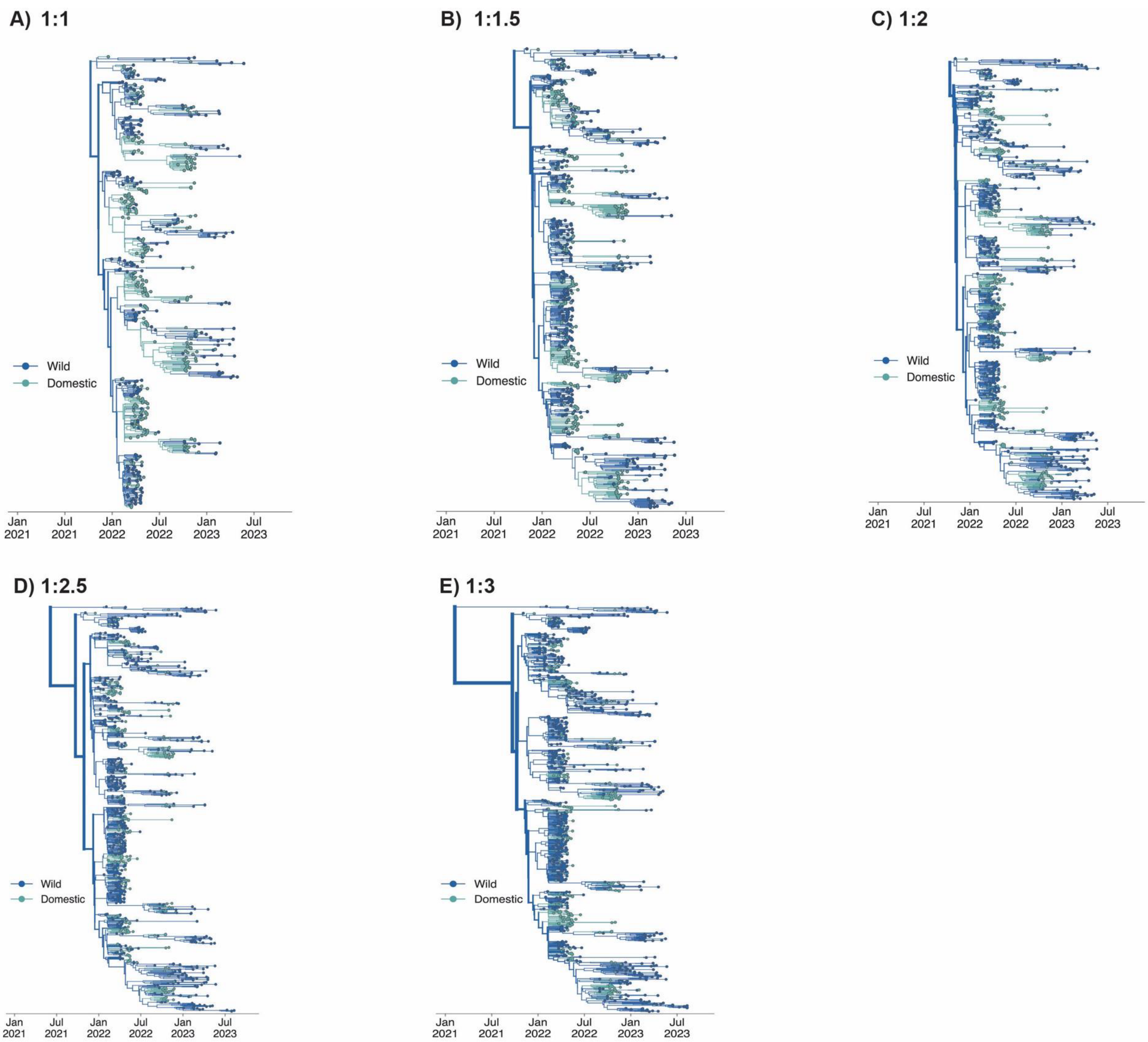

Figure S11: Two-state rarefaction analysis (domestic:wild MCC trees A-E) show the ratios of domestic to wild in increasing order of the number of wild bird sequences. Tips and branches are colored by the state (wild or domestic) of the sample and inferred state respectively.

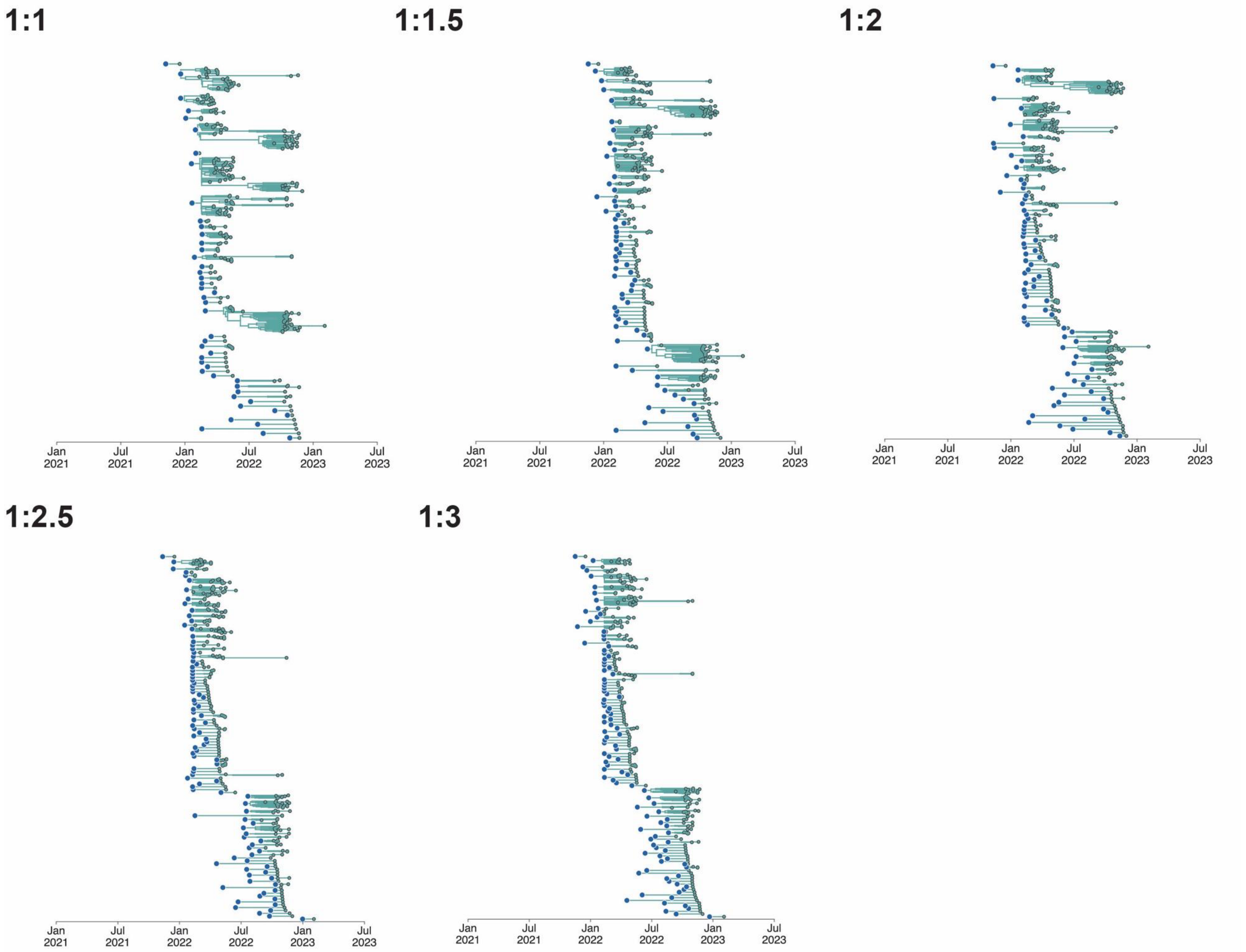

Figure S12: A) Exploded tree view of the MCC tree of two state rarefaction for wild to domestic transitions. Colors correspond to the state of a given tip, branch node.

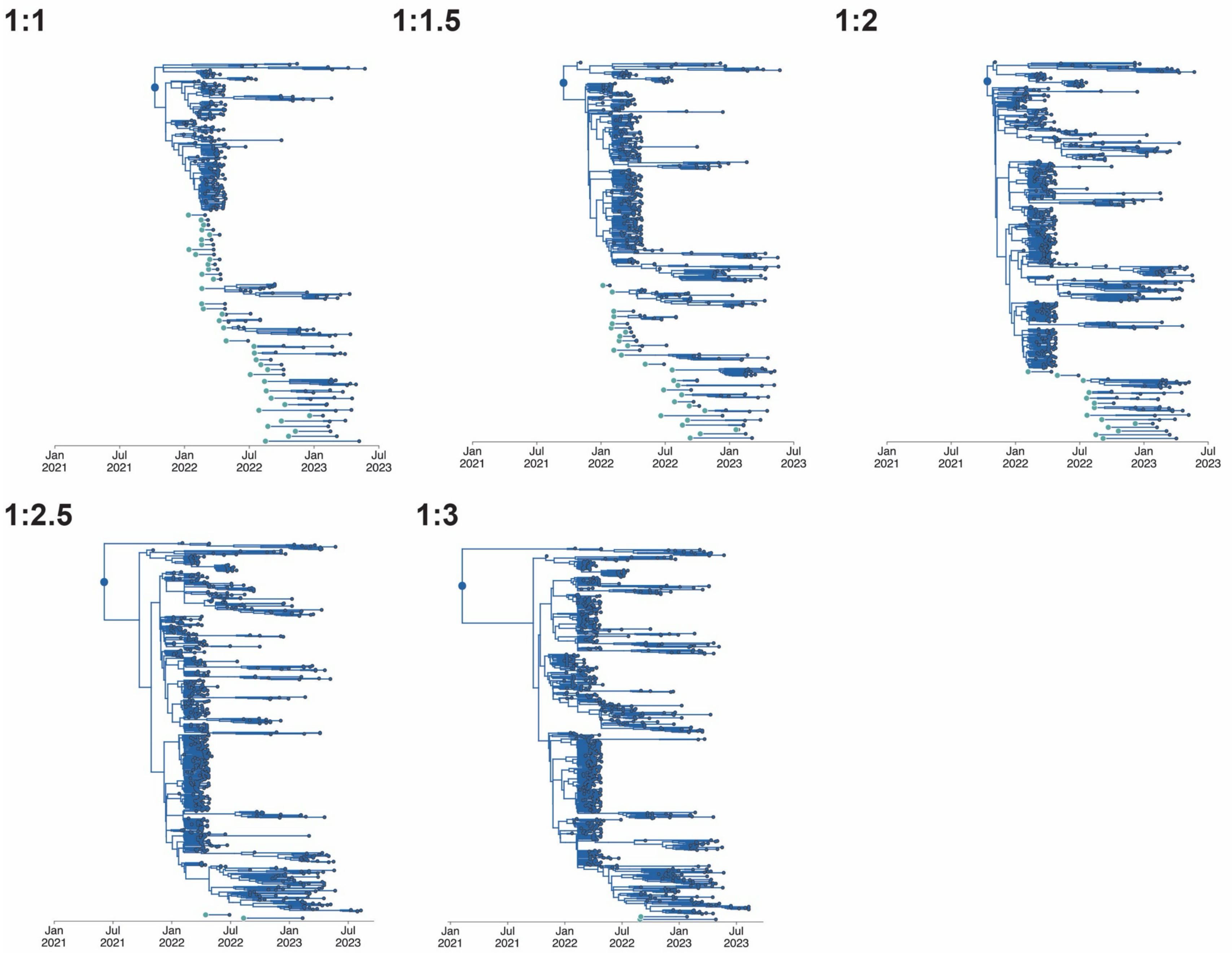

Figure S13: A) Exploded tree view of the MCC tree of two state rarefactions for domestic to wild transitions. Colors correspond to the state of a given tip, branch node.

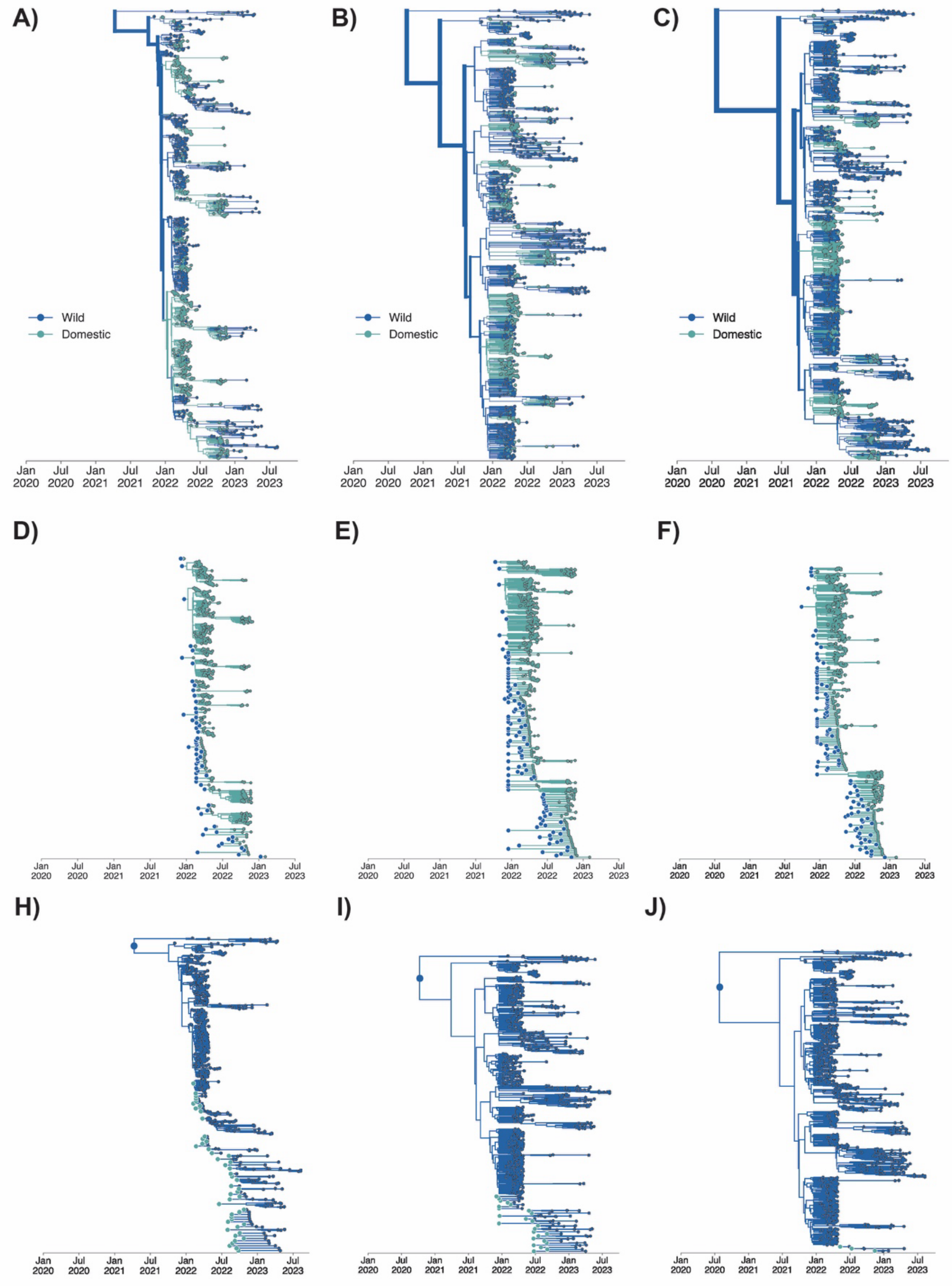

Figure S14: A-C) MCC trees of the ratios of domestic to wild including turkey sequences in increasing order of the number of wild bird sequences. Tips and branches are colored by the state (wild or domestic) of the sample and inferred state respectively. D-F) Exploded tree views of the MCC trees for each titration of two state rarefactions including turkey for wild to domestic transitions. G-J) Exploded tree views of the MCC trees for each titration of two state rarefactions including turkey for domestic to wild transitions. Colors correspond to the state of a given tip, branch node.

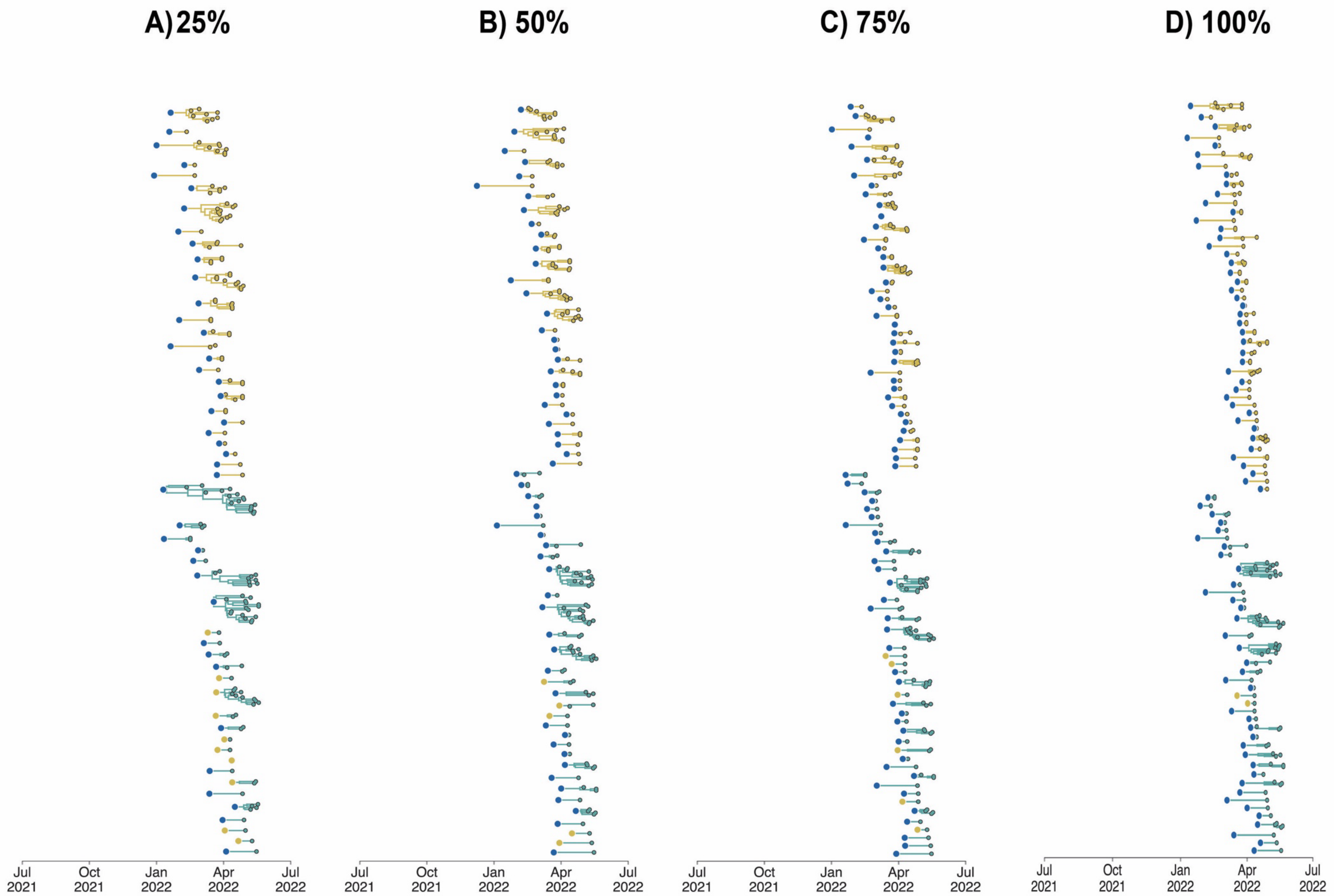

Figure S15: Exploded tree view of MCC trees for each tree in the three-state titration analysis. Percentage refers to the percentage of all available wild sequences used in the given analysis. Subtrees represent the traversal of a tree from the root to the tip where the state is unchanged from the initial state (given by the large dot on left) to the tips represented by the smaller dots representing continuous chains of transmission within a given state. Colors correspond to the state of a given tip, branch node where blue = wild bird, green = commercial bird, yellow = backyard bird.

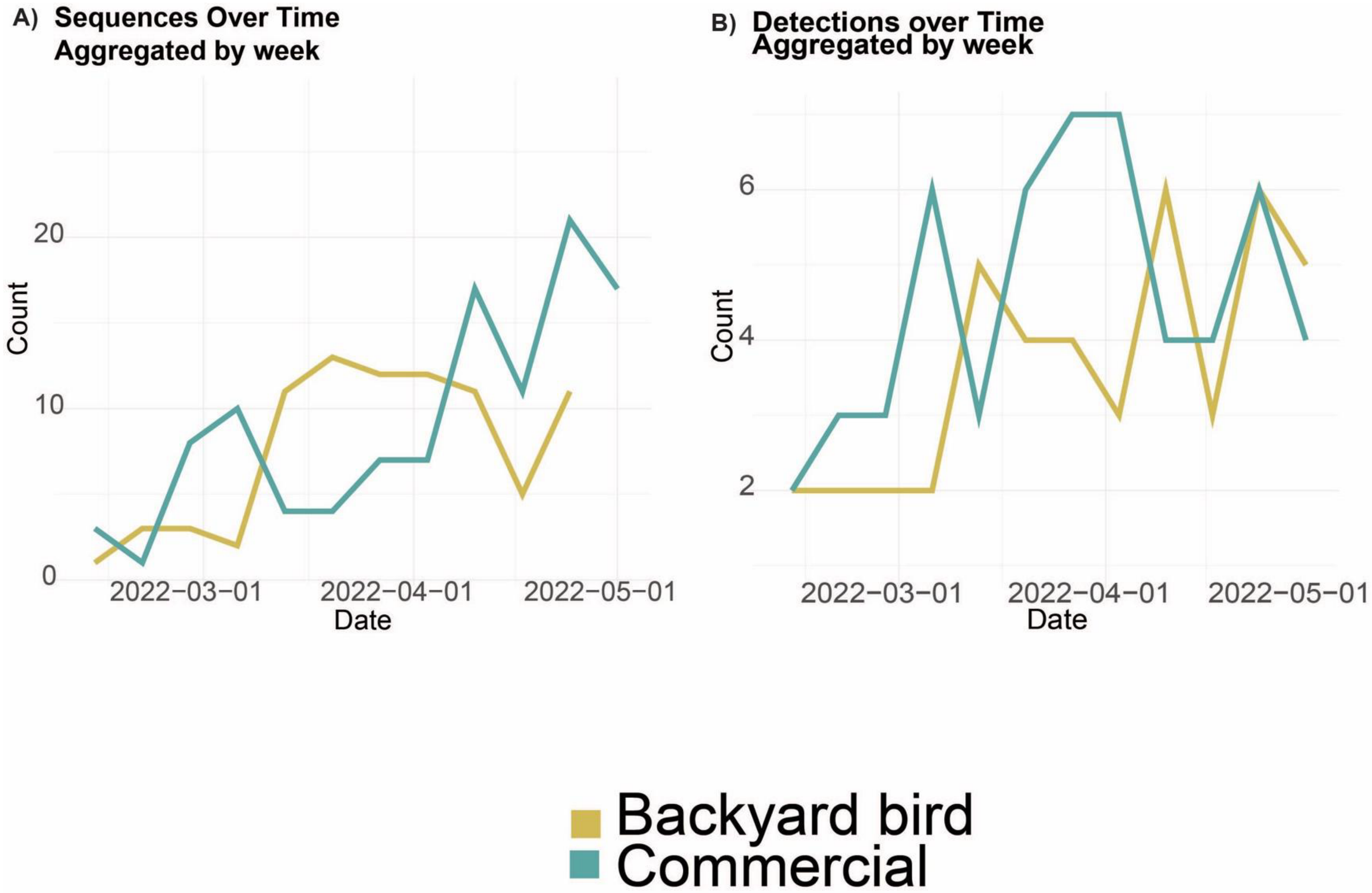

Figure S16. A) Number of sequences over time aggregated by week from Backyard birds (yellow) and commercial birds (Green) between 2022-02-12 and 2022-05-01. B) Number of detections (farms/premises) over time aggregated by week from WOAH Non-Poultry (Backyard birds) and WOAH Poultry (commercial birds) between 2022-02-12 and 2022-05-01. C) Number of times that a given record production type for a detection transitioned to a different production type for the next record in chronological order for all available detections.

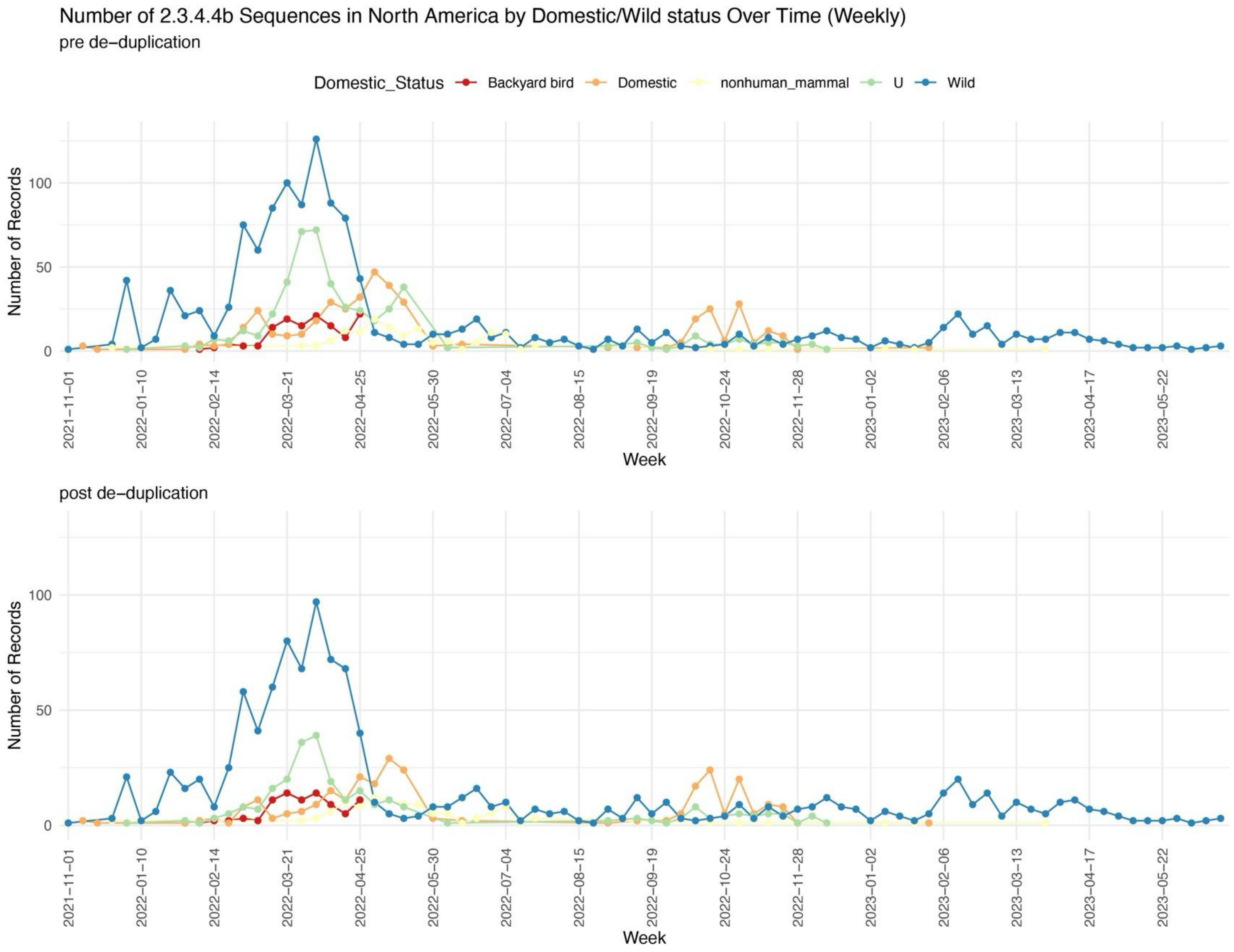

Figure S17. Number of sequences by week by domestic/wild status determined by available metadata before and after deduplication of identical sequences occurring on the same day. Post de-duplication total number of sequences 1818.

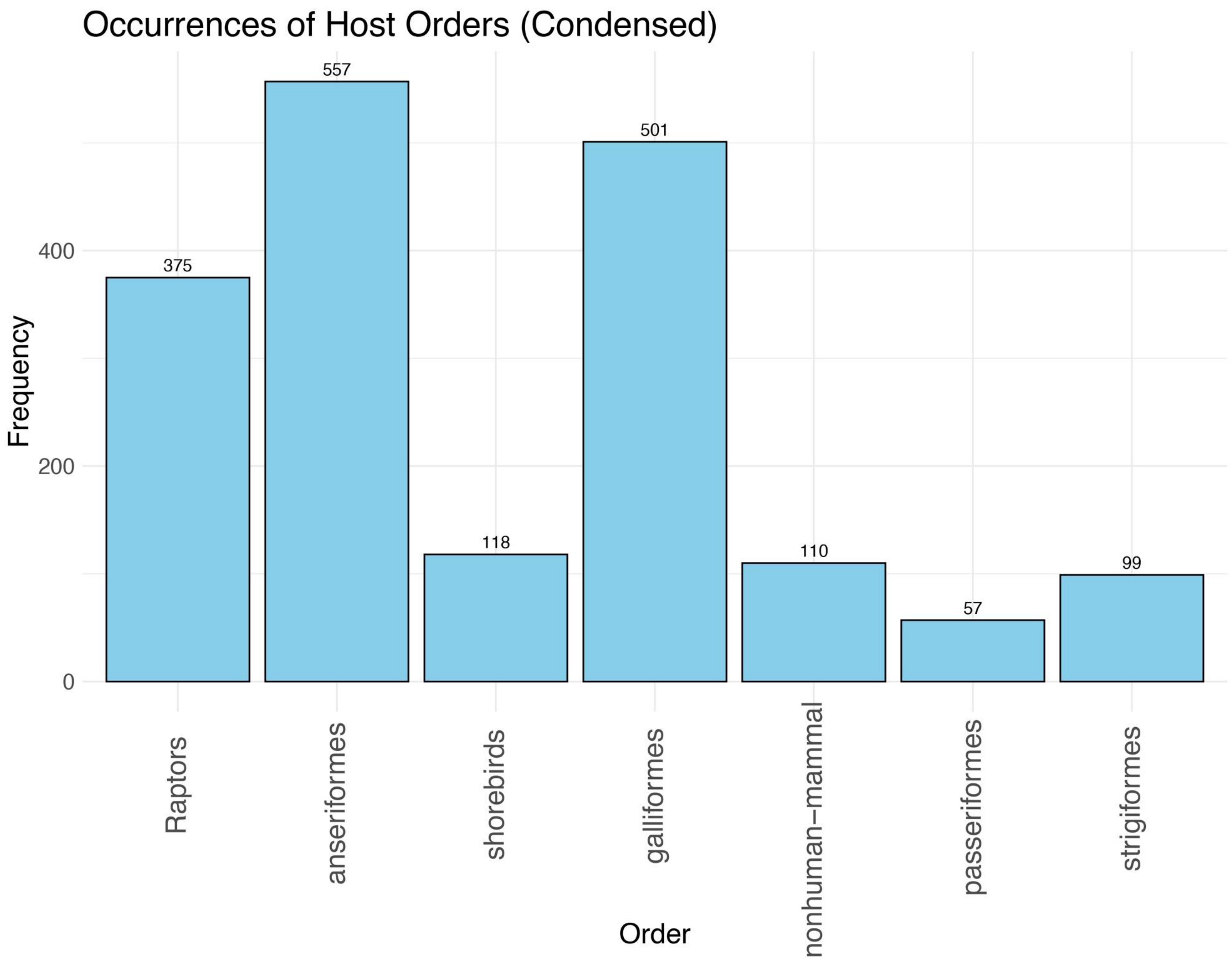

Figure S18. Number of sequences for each host taxonomic order for available sequence data.

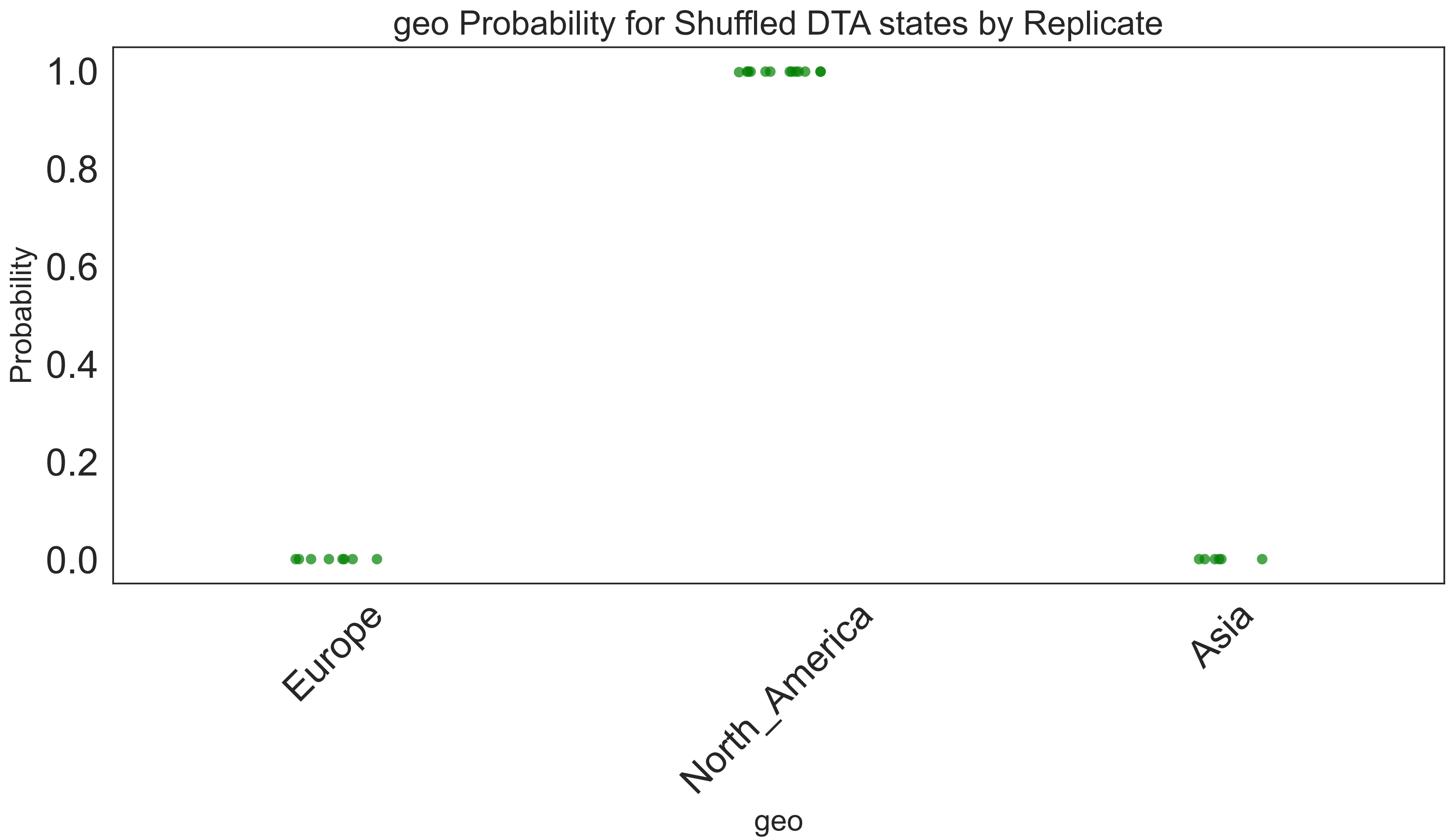

Figure S19 Root state probabilities for 100 replicates of tip shuffle test for Global introduction dataset.

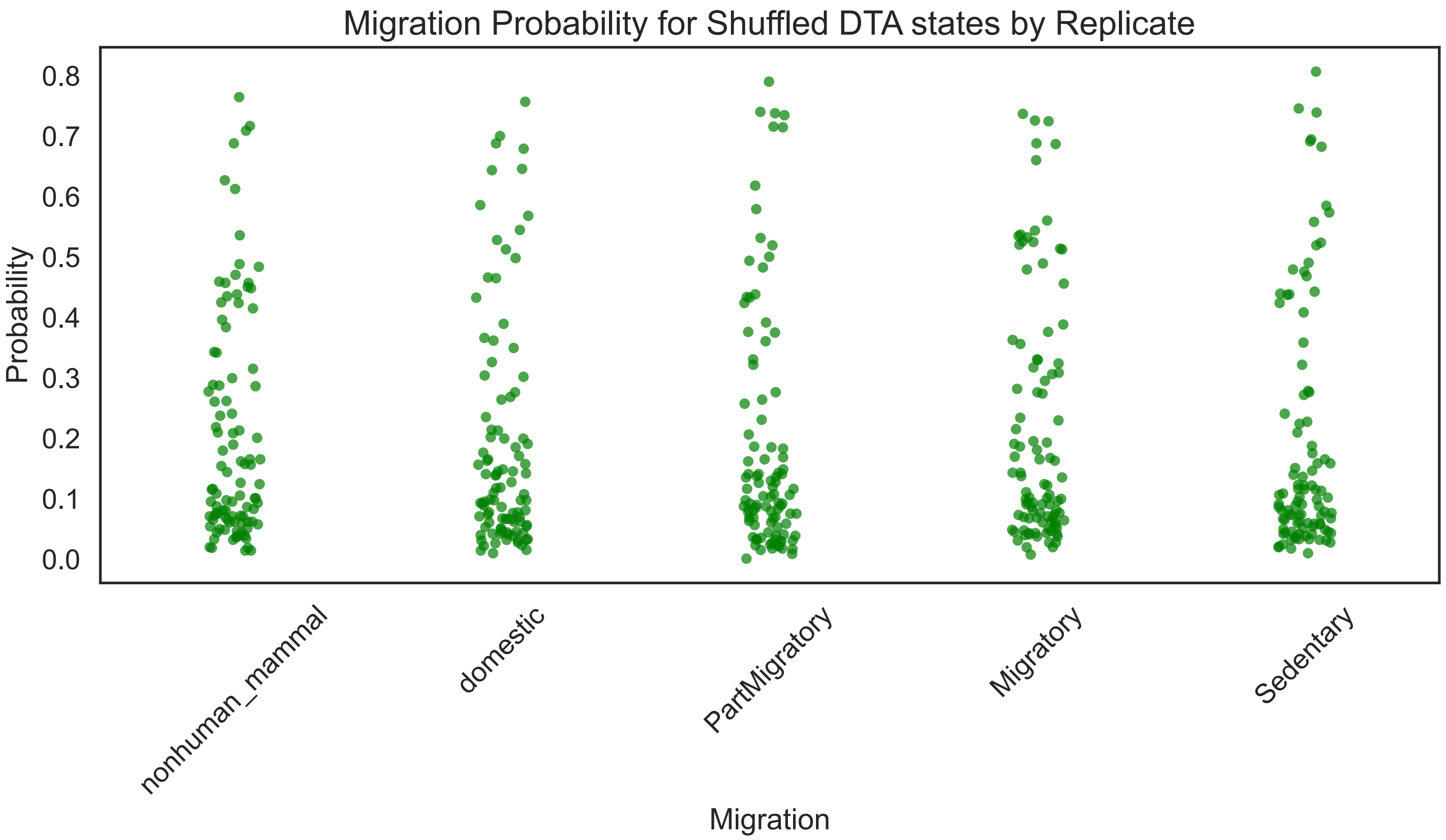

Figure S20 Root state probabilities for 100 replicates of tip shuffle test for Migratory behavior dataset.

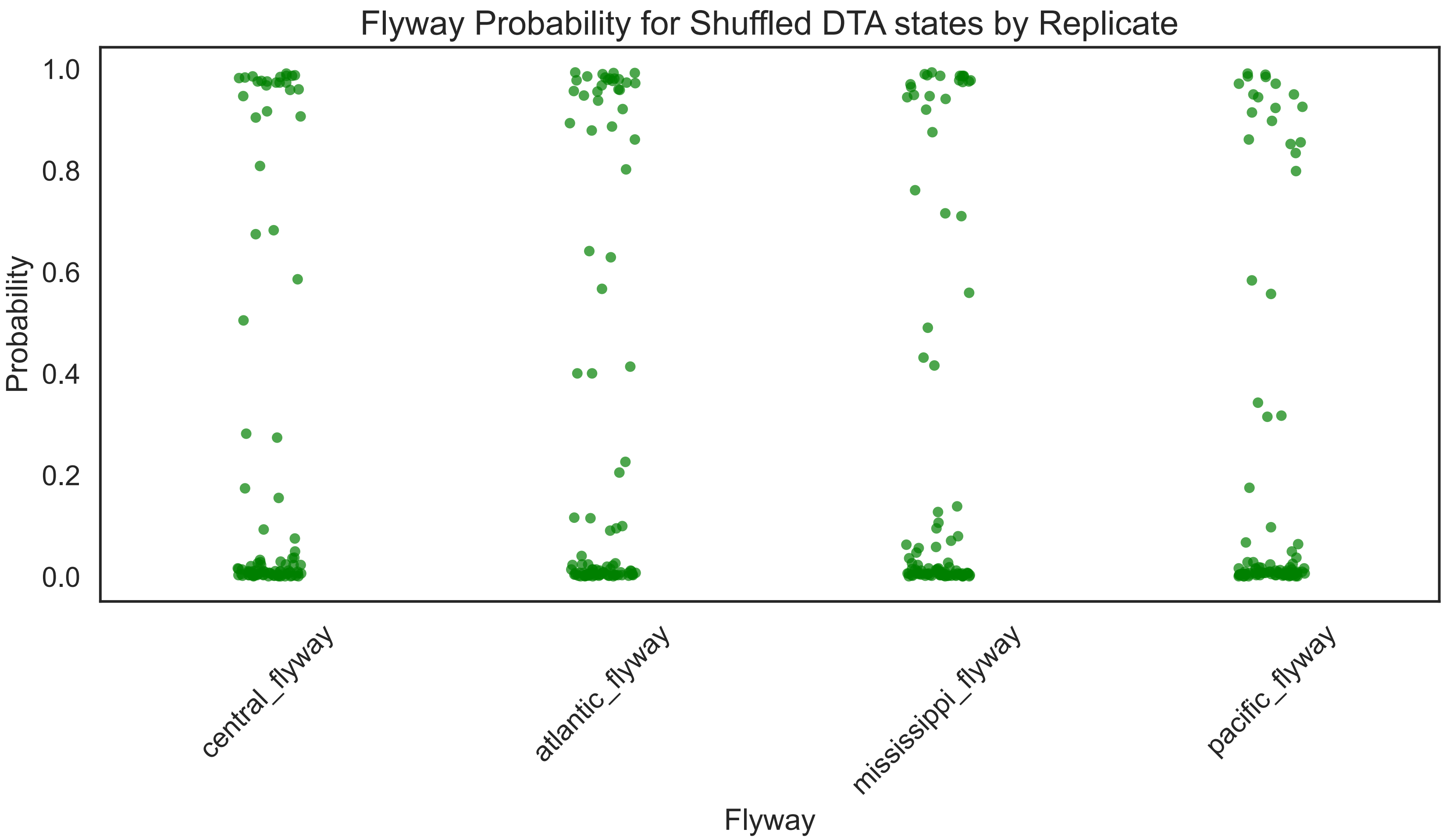

Figure S21 Root state probabilities for 100 replicates of tip shuffle test for flyway dataset.

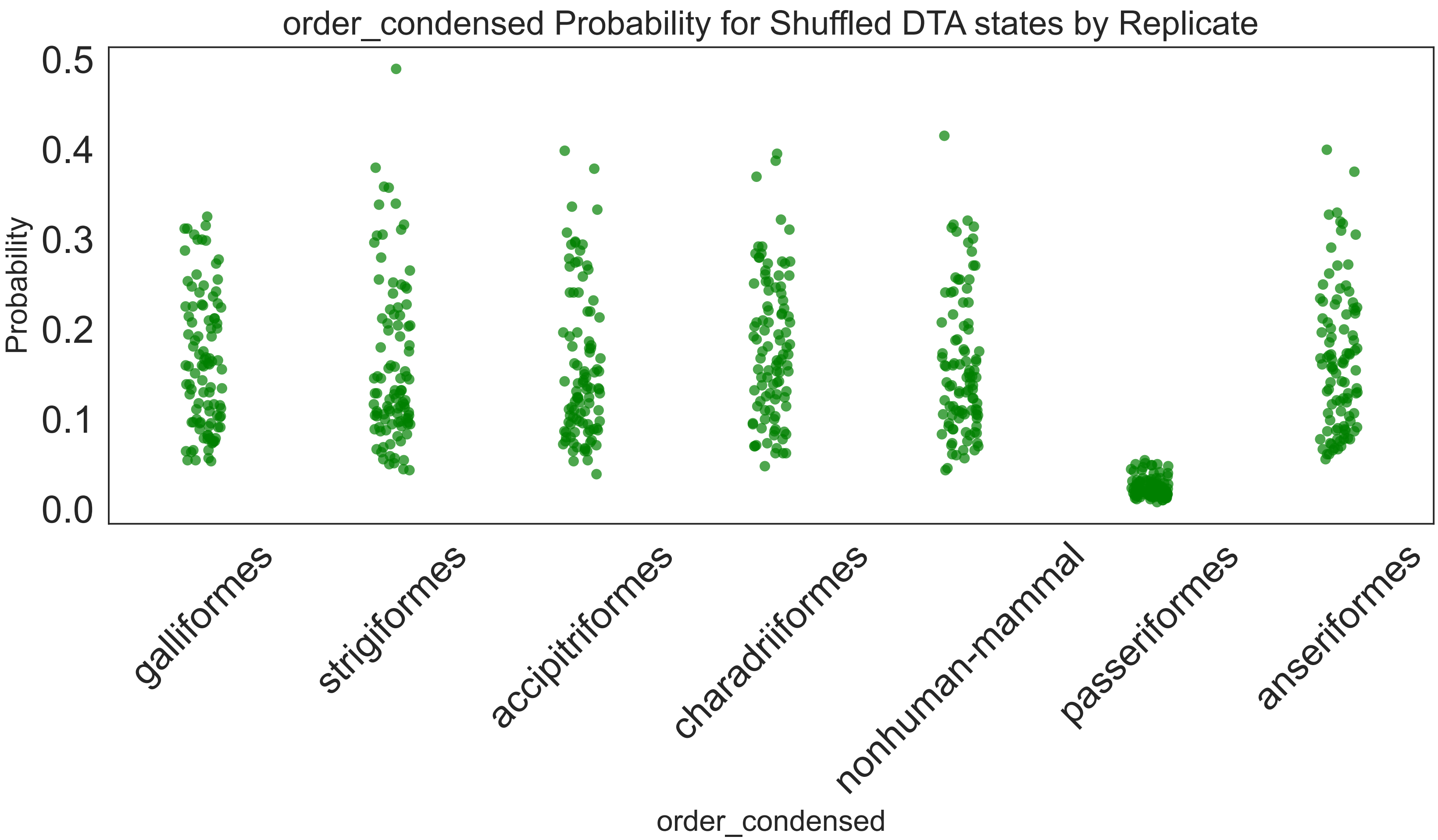

Figure S22 Root state probabilities for 100 replicates of tip shuffle test for order combined

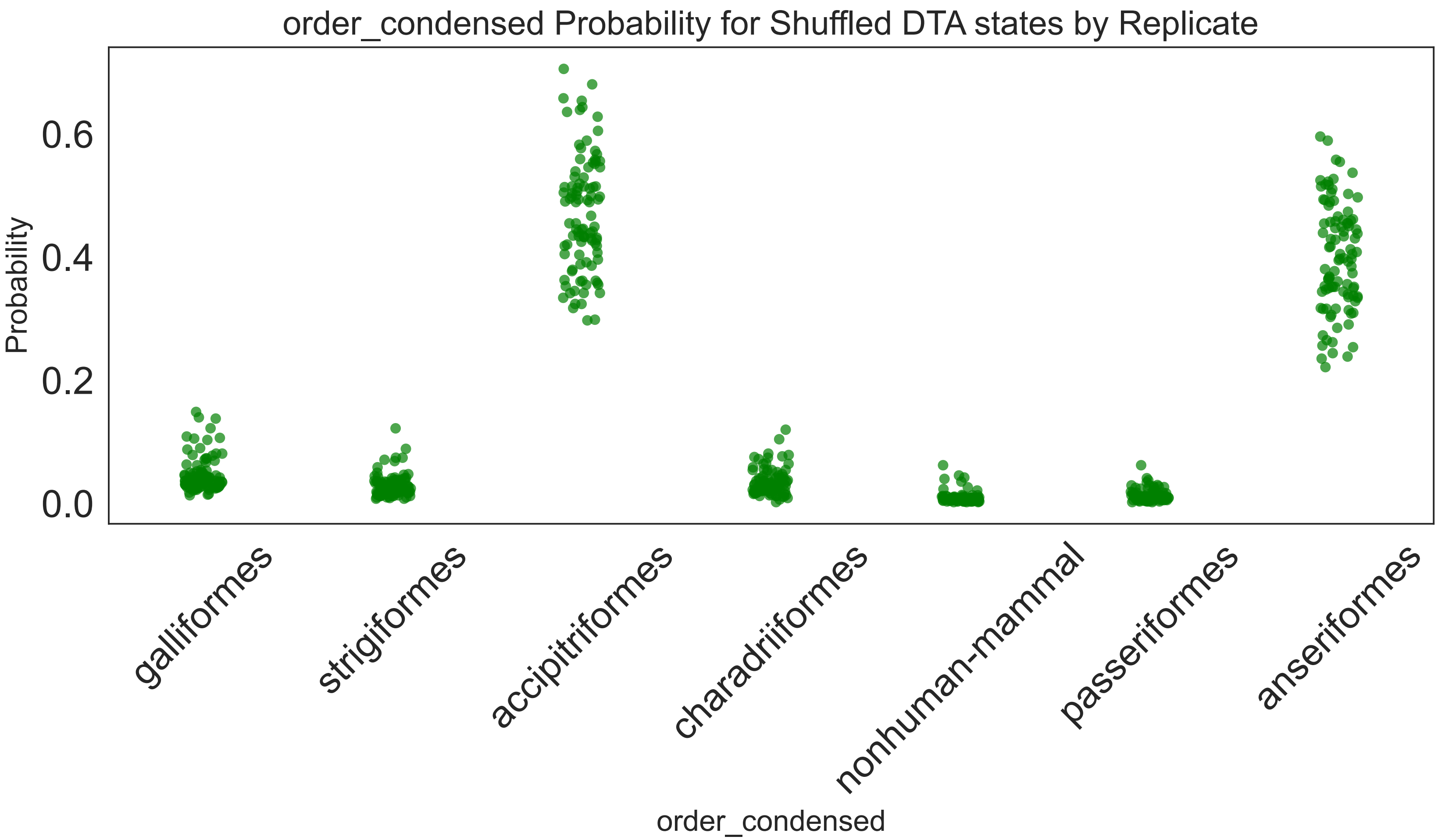

Figure S23 Root state probabilities for 100 replicates of tip shuffle test for order prop combined dataset.

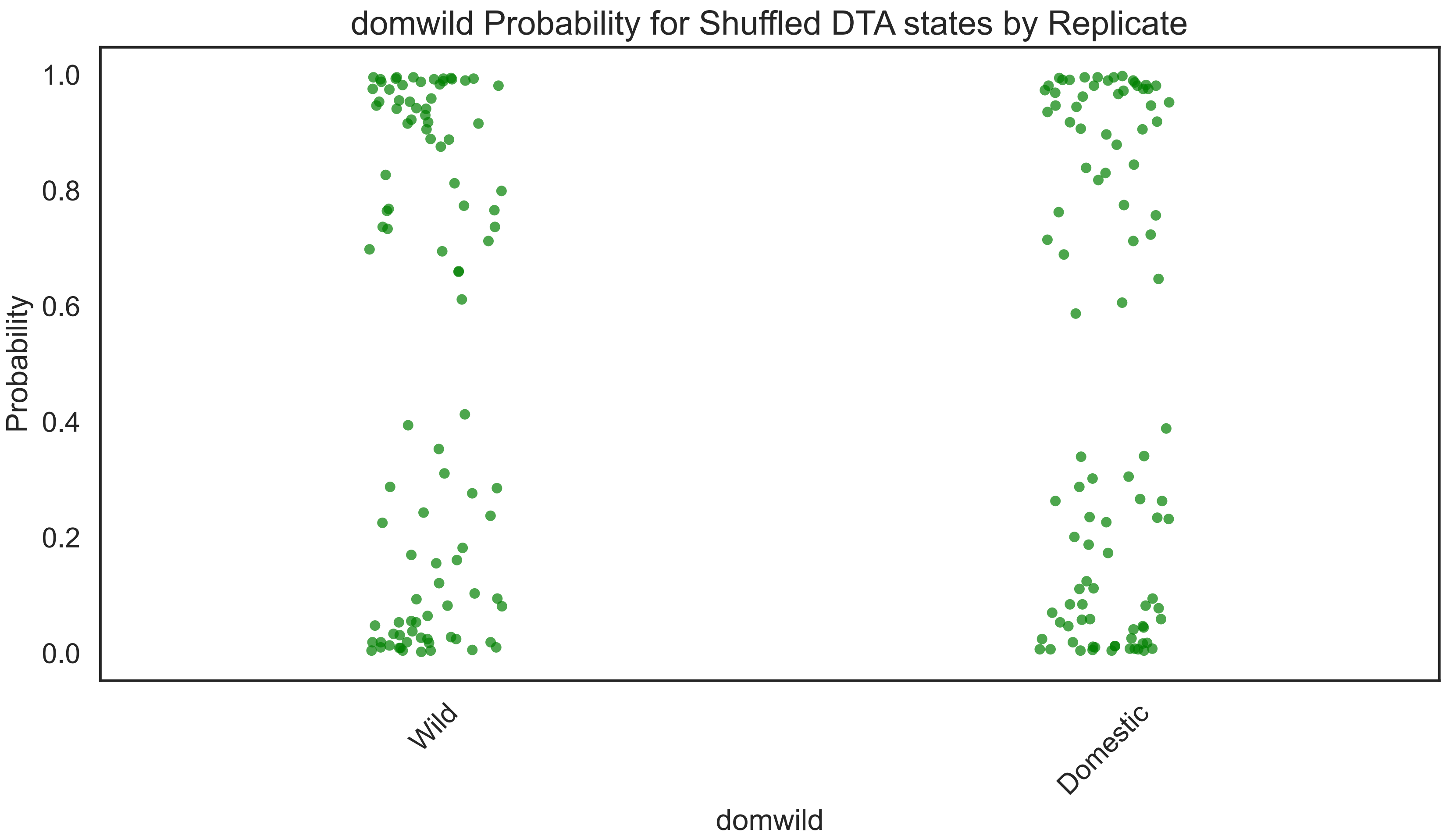

Figure S24 Root state probabilities for 100 replicates of tip shuffle test for domestic wild (1:1) dataset.

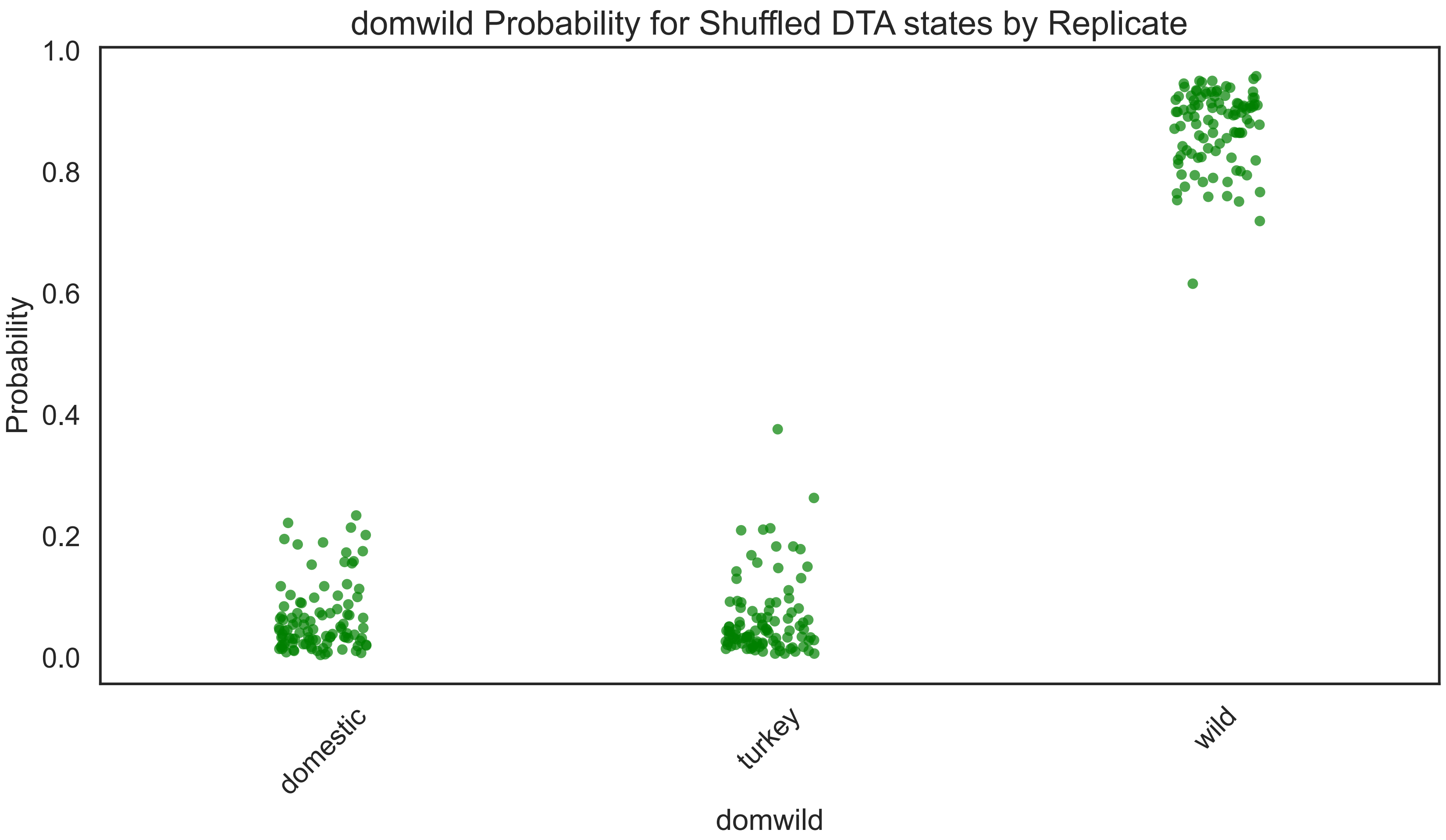

Figure S25 Root state probabilities for 100 replicates of tip shuffle test for domestic wild turkey (1:1:1) dataset.

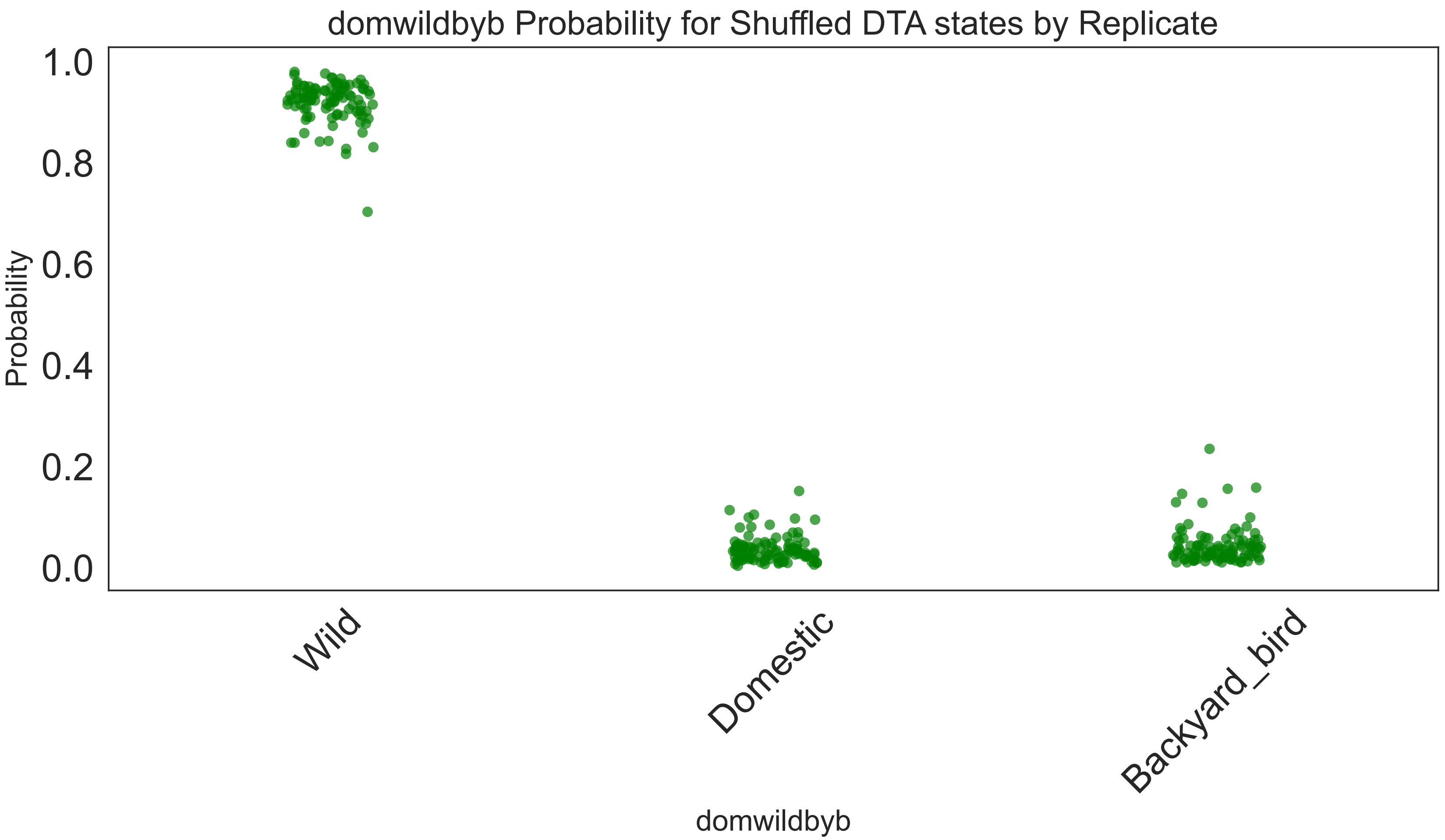

Figure S26 Root state probabilities for 100 replicates of tip shuffle test for domestic wild backyard bird dataset (1:1 domestic:backyard bird + 25% all wild).

Supplementary Tables

From	To	mean	median	l95hpd	h95hpd	BF	Post. prob	mean Markov Jumps	Markov Jumps 95% lhpd	Markov Jumps 95% hhpd	mean Markov Jump / tree height	Markov jumps / tree height lower 95%hpd	Markov jumps / tree height upper 95%hpd	Markov Jump / tree length	Markov Jump / tree length lower 95% hpd	Markov Jump / tree length upper 95% hpd
**Atlantic**	**Mississippi**	**2.305**	**2.186**	**0.742**	**4.08**	**27000**	**1**	**70.232**	**58**	**81**	**37.34**	**30.837**	**43.065**	**0.345**	**0.285**	**0.398**
Atlantic	Pacific	0.505	0.223	0.004	2.349	9.672	0.763	3.163	1	5	1.681	0.532	2.658	0.016	0.005	0.025
**Central**	**Pacific**	**1.935**	**1.823**	**0.641**	**3.48**	**27000**	**1**	**24.69**	**15**	**34**	**13.127**	**7.975**	**18.077**	**0.121**	**0.074**	**0.167**
**Central**	**Mississippi**	**0.899**	**0.818**	**0.21**	**1.746**	**27000**	**1**	**16.657**	**10**	**23**	**8.856**	**5.317**	**12.228**	**0.082**	**0.049**	**0.113**
**Mississippi**	**Central**	**4.687**	**4.491**	**1.67**	**7.873**	**27000**	**1**	**105.895**	**90**	**121**	**56.301**	**47.85**	**64.332**	**0.52**	**0.442**	**0.595**
**Mississippi**	**Atlantic**	**0.724**	**0.643**	**0.111**	**1.529**	**95.912**	**0.97**	**2.689**	**1**	**7**	**1.43**	**0.532**	**3.722**	**0.013**	**0.005**	**0.034**
Pacific	Central	0.698	0.627	0.139	1.418	27000	1	21.134	15	25	11.236	7.975	13.292	0.104	0.074	0.123

Table S1: Results of BSSVS discrete trait analysis for USFWS flyways. Table is in order of highest mean transition rate to lowest with rates having BF > 100 bolded. Rates with at least a BF > 3 are shown.

From	To	mean	median	l95hpd	h95hpd	BAYES_FACTOR	POSTERIOR PROBABILITY
**group1_combined_above49N**	**group2_northernus_southerncanada_42N_49N**	**0.72298544**	**0.66041858**	**0.14856531**	**1.39344548**	**29700**	**1**
**group1_combined_above49N**	**group4_southernus_below36N**	**0.86767129**	**0.80234959**	**0.20925934**	**1.6251426**	**29700**	**1**
**group3_centralus_36N_42N**	**group2_northernus_southerncanada_42N_49N**	**1.82410787**	**1.69980647**	**0.48516246**	**3.46284291**	**29700**	**1**
**group4_southernus_below36N**	**group2_northernus_southerncanada_42N_49N**	**0.82898677**	**0.76352712**	**0.19578435**	**1.64072213**	**29700**	**1**
**group4_southernus_below36N**	**group3_centralus_36N_42N**	**1.92837598**	**1.82155582**	**0.63907759**	**3.45480903**	**29700**	**1**
**group2_northernus_southerncanada_42N_49N**	**group3_centralus_36N_42N**	**1.31353336**	**1.20124055**	**0.26230058**	**2.59867265**	**9898**	**0.999697**
**group2_northernus_southerncanada_42N_49N**	**group1_combined_above49N**	**1.05557566**	**0.97455431**	**0.23064274**	**2.03878799**	**799.783784**	**0.996263**
group2_northernus_southerncanada_42N_49N	group4_southernus_below36N	0.60581523	0.53262685	0.04471526	1.32288619	86.7371601	0.96656903
group3_centralus_36N_42N	group1_combined_above49N	0.52887832	0.3500056	0.00020955	1.69816282	7.69992795	0.71962428
group3_centralus_36N_42N	group4_southernus_below36N	0.76006982	0.4825803	2.47E-05	2.51017546	2.44809244	0.44934855
group1_combined_above49N	group3_centralus_36N_42N	0.65898606	0.34038264	2.89E-05	2.39739077	2.28899573	0.43278457
group4_southernus_below36N	group1_combined_above49N	0.85125837	0.51546685	4.59E-05	2.79417744	0.59513435	0.16553883

Table S2: Results of BSSVS discrete trait analysis for geographic group based on latitude. Table is in order of highest mean transition rate to lowest with rates having BF > 100 bolded. Rates with at least a BF > 3 are shown.

From	To	BAYES_FACTOR	POSTERIOR PROBABILITY	mean	median	l95hpd	h95hpd
**Migration**	**PartMigration**	**36000**	**1**	**2.00874182**	**1.90919107**	**0.73749557**	**3.48482027**
**Migration**	**Sedentary**	**181.587629**	**0.97844684**	**1.04729841**	**0.95439458**	**0.11917316**	**2.10127971**
**Migration**	**Domestic**	**36000**	**1**	**1.33497394**	**1.25320356**	**0.40376184**	**2.38054574**
**Migration**	**Nonhumanmammal**	**400.539326**	**0.99011221**	**1.19631674**	**1.12017969**	**0.24933207**	**2.33312444**
PartMigration	Sedentary	97.9943343	0.96078214	0.87098186	0.77876747	0.07788287	1.84752126
PartMigration	Domestic	0.52141153	0.11532052	0.91600762	0.58774557	0.00061945	2.89768449
PartMigration	Nonhumanmammal	1.13316225	0.22075325	0.8826551	0.55567327	0.00047205	2.81096245
Sedentary	Domestic	0.53450882	0.11787579	0.95396382	0.62897596	0.00061559	2.96816781
Sedentary	Nonhumanmammal	3.036154	0.43150761	0.779748	0.52251605	3.72E-06	2.41343409
Domestic	Nonhumanmammal	20.3929539	0.83601822	0.93032214	0.80834525	0.01262889	2.11755253
PartMigration	Migration	2.21508717	0.35640484	0.75888425	0.4470211	0.0005076	2.5426762
Sedentary	Migration	36000	1	1.44051268	1.35975899	0.48396807	2.58706142
Domestic	Migration	1.43128677	0.26352628	0.83338968	0.52381554	5.85E-05	2.68385089
Nonhumanmammal	Migration	0.50275138	0.11165426	0.89103885	0.58370738	8.10E-05	2.77166192
Sedentary	PartMigration	5.62416466	0.58437951	0.7707354	0.57152876	5.10E-05	2.17327905
Domestic	PartMigration	10.9891757	0.73314076	0.75017909	0.58957738	0.00020665	1.96900017
Nonhumanmammal	PartMigration	140.594378	0.97233641	0.89119182	0.80402919	0.03583859	1.84688931
Domestic	Sedentary	4.18272727	0.51116543	0.88474002	0.68014107	0.00151883	2.41678285
Nonhumanmammal	Sedentary	510.342857	0.99222309	1.19024247	1.11177603	0.24962462	2.29915023
Nonhumanmammal	Domestic	4.69661836	0.54005111	0.64654853	0.41283259	0.00046807	2.10029534

Table S3: Results of BSSVS discrete trait analysis for Migratory Behavior. Table is in order of highest mean transition rate to lowest with rates having BF > 100 bolded. Rates with at least a BF > 3 are shown.

From	To	mean	median	l95hpd	h95hpd	BAYES FACTOR	POSTERIOR PROB	mean Markov Jumps	Markov jumps lower 95%hpd	Markov jumps upper 95%hpd	Markov Jumps / tree height	Markov jumps / tree hieght lower 95%hpd	Markov jumps / tree hiegh t upper 95% hpd	Markov Jump / tree length	Markov Jump / tree length lower 95% hpd	Markov Jump / tree length upper 95% hpd
**Anseriformes**	**Galliformes**	**4.494**	**4.374**	**1.842**	**7.208**	**1691.250**	**0.996**	**49.950**	**26.000**	**73.000**	**17.808**	**9.269**	**26.025**	**0.493**	**0.257**	**0.721**
**Shorebirds**	**Nonhuman-Mammal**	**2.089**	**1.970**	**0.640**	**3.620**	**537.120**	**0.989**	**17.035**	**9.000**	**25.000**	**6.073**	**3.209**	**8.913**	**0.168**	**0.089**	**0.247**
**Shorebirds**	**Raptors**	**1.787**	**1.668**	**0.358**	**3.298**	**245.444**	**0.976**	**14.651**	**5.000**	**24.000**	**5.223**	**1.783**	**8.556**	**0.145**	**0.049**	**0.237**
**Anseriformes**	**Strigiformes**	**3.406**	**3.238**	**1.239**	**6.141**	**232.211**	**0.975**	**37.889**	**15.000**	**64.000**	**13.508**	**5.348**	**22.816**	**0.374**	**0.148**	**0.632**
**Galliformes**	**Strigiformes**	**3.015**	**2.871**	**0.853**	**5.365**	**193.676**	**0.970**	**28.517**	**4.000**	**47.000**	**10.166**	**1.426**	**16.756**	**0.282**	**0.039**	**0.464**
**Galliformes**	**Nonhuman-Mammal**	**2.648**	**2.524**	**0.779**	**4.771**	**170.338**	**0.966**	**24.653**	**5.000**	**38.000**	**8.789**	**1.783**	**13.547**	**0.243**	**0.049**	**0.375**
**Shorebirds**	**Passeriformes**	**1.219**	**1.103**	**0.268**	**2.428**	**150.069**	**0.962**	**9.351**	**3.000**	**14.000**	**3.334**	**1.070**	**4.991**	**0.092**	**0.030**	**0.138**
**Galliformes**	**Anseriformes**	**2.472**	**2.296**	**0.453**	**4.882**	**146.562**	**0.961**	**22.044**	**2.000**	**42.000**	**7.859**	**0.713**	**14.973**	**0.218**	**0.020**	**0.415**
**Anseriformes**	**Shorebirds**	**1.775**	**1.662**	**0.330**	**3.411**	**143.209**	**0.960**	**18.978**	**5.000**	**34.000**	**6.766**	**1.783**	**12.121**	**0.187**	**0.049**	**0.336**
**Anseriformes**	**Raptors**	**2.527**	**2.344**	**0.383**	**4.865**	**127.118**	**0.955**	**27.702**	**6.000**	**51.000**	**9.876**	**2.139**	**18.182**	**0.273**	**0.059**	**0.504**
**Galliformes**	**Passeriformes**	**2.043**	**1.967**	**0.469**	**3.785**	**117.436**	**0.951**	**18.563**	**5.000**	**30.000**	**6.618**	**1.783**	**10.695**	**0.183**	**0.049**	**0.296**
Strigiformes	Passeriformes	1.574	1.455	0.183	3.095	81.600	0.932	11.313	2.000	20.000	4.033	0.713	7.130	0.112	0.020	0.197
Raptors	Nonhuman-Mammal	1.797	1.633	0.240	3.673	71.148	0.922	14.656	3.000	27.000	5.225	1.070	9.626	0.145	0.030	0.267
Strigiformes	Raptors	1.919	1.753	0.235	3.942	61.218	0.911	13.627	3.000	25.000	4.858	1.070	8.913	0.135	0.030	0.247
Nonhuman-Mammal	Anseriformes	2.236	2.039	0.207	4.517	53.815	0.900	15.836	2.000	29.000	5.646	0.713	10.339	0.156	0.020	0.286
Raptors	Strigiformes	1.710	1.539	0.178	3.444	51.534	0.896	13.861	2.000	25.000	4.941	0.713	8.913	0.137	0.020	0.247
Raptors	Shorebirds	1.681	1.555	0.169	3.486	47.881	0.889	13.376	1.000	24.000	4.769	0.357	8.556	0.132	0.010	0.237
Galliformes	Raptors	2.009	1.857	0.195	4.059	41.642	0.874	19.161	1.000	35.000	6.831	0.357	12.478	0.189	0.010	0.346
Raptors	Anseriformes	1.873	1.719	0.182	3.942	38.518	0.865	14.530	1.000	26.000	5.180	0.357	9.269	0.143	0.010	0.257
Galliformes	Shorebirds	1.479	1.311	0.129	3.152	33.818	0.849	12.235	2.000	23.000	4.362	0.713	8.200	0.121	0.020	0.227
Shorebirds	Anseriformes	1.209	0.952	0.013	3.149	17.014	0.739	7.472	1.000	15.000	2.664	0.357	5.348	0.074	0.010	0.148
Strigiformes	Shorebirds	1.487	1.187	0.016	3.692	11.588	0.659	9.110	1.000	18.000	3.248	0.357	6.417	0.090	0.010	0.178
Strigiformes	Nonhuman-Mammal	1.321	1.040	0.005	3.421	10.660	0.640	7.089	1.000	14.000	2.527	0.357	4.991	0.070	0.010	0.138
Raptors	Galliformes	1.314	1.030	0.002	3.302	9.807	0.620	8.312	1.000	17.000	2.963	0.357	6.061	0.082	0.010	0.168
Nonhuman-Mammal	Galliformes	1.553	1.262	0.009	3.804	8.727	0.593	7.869	1.000	16.000	2.805	0.357	5.704	0.078	0.010	0.158
Anseriformes	Nonhuman-Mammal	1.574	1.334	0.010	3.769	8.399	0.583	16.845	1.000	45.000	6.005	0.357	16.043	0.166	0.010	0.444

Passeriformes	Raptors	1.494	1.206	0.006	3.750	8.353	0.582	5.655	1.000	12.000	2.016	0.357	4.278	0.056	0.010	0.118
Nonhuman-Mammal	Shorebirds	1.369	1.048	0.009	3.769	6.080	0.503	6.318	1.000	14.000	2.252	0.357	4.991	0.062	0.010	0.138
Nonhuman-Mammal	Raptors	1.383	1.076	0.004	3.619	6.069	0.503	6.761	1.000	15.000	2.410	0.357	5.348	0.067	0.010	0.148
Nonhuman-Mammal	Strigiformes	1.370	1.037	0.002	3.771	6.059	0.502	6.575	1.000	14.000	2.344	0.357	4.991	0.065	0.010	0.138
Passeriformes	Anseriformes	1.512	1.156	0.037	4.284	5.963	0.498	5.226	1.000	11.000	1.863	0.357	3.922	0.052	0.010	0.109
Strigiformes	Galliformes	1.434	1.136	0.005	3.733	5.487	0.478	8.253	1.000	18.000	2.942	0.357	6.417	0.081	0.010	0.178
Anseriformes	Passeriformes	1.441	1.124	0.014	3.748	5.066	0.458	13.073	1.000	28.000	4.660	0.357	9.982	0.129	0.010	0.276

Table S4: Results of BSSVS discrete trait analysis for combined results of equal host schema. Table is in order of highest mean transition rate to lowest with rates having BF > 100 bolded. Rates with at least a BF > 3 are shown.

From	To	mean	median	l95hpd	h95hpd	BAYES FACTOR	POSTERIOR PROBABILITY	mean Markov Jumps	Markov Jumps 95% lhpd	Markov Jumps 95% hhpd	mean Markov Jump / tree height	Markov jumps / tree hieght lower 95%hpd	Markov jumps / tree hieght upper 95%hpd	Markov Jump / tree length	Markov Jump / tree length lower 95% hpd	Markov Jump / tree length upper 95% hpd
**Anseriformes**	**Galliformes**	**4.427**	**4.291**	**2.201**	**6.781**	**18006.000**	**1.000**	**47.738**	**30.000**	**61.000**	**21.275**	**13.370**	**27.186**	**0.345**	**0.217**	**0.441**
**Anseriformes**	**Nonhuman-Mammal**	**0.911**	**0.856**	**0.183**	**1.659**	**321.491**	**0.982**	**12.112**	**1.000**	**27.000**	**5.398**	**0.446**	**12.033**	**0.088**	**0.007**	**0.195**
**Anseriformes**	**Passeriformes**	**0.680**	**0.626**	**0.158**	**1.296**	**714.480**	**0.992**	**14.041**	**5.000**	**23.000**	**6.258**	**2.228**	**10.250**	**0.102**	**0.036**	**0.166**
**Anseriformes**	**Raptors**	**5.885**	**5.711**	**2.805**	**9.178**	**18006.000**	**1.000**	**85.675**	**69.000**	**99.000**	**38.183**	**30.751**	**44.122**	**0.620**	**0.499**	**0.716**
**Anseriformes**	**Shorebirds**	**1.647**	**1.565**	**0.359**	**3.094**	**1379.538**	**0.996**	**25.412**	**11.000**	**43.000**	**11.325**	**4.902**	**19.164**	**0.184**	**0.080**	**0.311**
**Anseriformes**	**Strigiformes**	**1.508**	**1.436**	**0.594**	**2.531**	**18006.000**	**1.000**	**27.070**	**12.000**	**40.000**	**12.064**	**5.348**	**17.827**	**0.196**	**0.087**	**0.289**
Galliformes	Anseriformes	1.391	1.101	0.002	3.668	6.371	0.515	7.856	1.000	19.000	3.501	0.446	8.468	0.057	0.007	0.138
Galliformes	Nonhuman-Mammal	1.131	0.842	0.001	3.323	3.880	0.393	4.239	1.000	14.000	1.889	0.446	6.239	0.031	0.007	0.101
Galliformes	Raptors	1.241	0.924	0.000	3.456	3.555	0.372	8.394	1.000	18.000	3.741	0.446	8.022	0.061	0.007	0.130
Passeriformes	Raptors	1.106	0.775	0.002	3.219	3.627	0.377	4.334	1.000	10.000	1.931	0.446	4.457	0.031	0.007	0.072
**Raptors**	**Anseriformes**	**2.951**	**2.753**	**0.880**	**5.633**	**299.288**	**0.980**	**26.316**	**3.000**	**54.000**	**11.728**	**1.337**	**24.066**	**0.190**	**0.022**	**0.391**
Raptors	Passeriformes	1.151	0.744	0.001	3.658	3.897	0.394	11.379	2.000	22.000	5.071	0.891	9.805	0.082	0.014	0.159
Raptors	Shorebirds	1.455	1.265	0.012	3.293	11.253	0.652	18.281	7.000	36.000	8.147	3.120	16.044	0.132	0.051	0.261
Raptors	Strigiformes	1.210	0.998	0.005	2.960	13.121	0.686	19.129	6.000	29.000	8.525	2.674	12.925	0.138	0.043	0.210
Shorebirds	Anseriformes	1.200	0.941	0.005	3.160	9.474	0.612	11.610	1.000	25.000	5.174	0.446	11.142	0.084	0.007	0.181
Shorebirds	Raptors	1.226	0.904	0.000	3.462	3.199	0.348	8.708	1.000	22.000	3.881	0.446	9.805	0.063	0.007	0.159

Table S5: Results of BSSVS discrete trait analysis for combined results of proportional schema. Table is in order of highest mean transition rate to lowest with rates having BF > 100 bolded. Rates with at least a BF > 3 are shown.

From	To	mean	median	l95hpd	h95hpd	BAYES FACTOR	POSTERIOR PROBABILITY	mean Markov Jumps	Markov Jumps 95% lhpd	Markov Jumps 95% hhpd	mean Markov Jump / tree height	Markov jumps / tree hieght lower 95%hpd	Markov jumps / tree hieght upper 95%hpd	Markov Jump / tree length	Markov Jump / tree length lower 95% hpd	Markov Jump / tree length upper 95% hpd
**Anseriformes**	**Galliformes**	**4.267**	**4.138**	**1.981**	**6.494**	**702.000**	**0.992**	**69.609**	**53.000**	**81.000**	**24.315**	**18.513**	**28.294**	**0.674**	**0.513**	**0.784**
**Anseriformes**	**Strigiformes**	**3.977**	**3.748**	**1.408**	**6.777**	**702.000**	**0.992**	**64.918**	**48.000**	**78.000**	**22.676**	**16.767**	**27.246**	**0.629**	**0.465**	**0.755**
**Galliformes**	**Strigiformes**	**2.863**	**2.712**	**0.467**	**4.893**	**151.333**	**0.962**	**8.935**	**1.000**	**19.000**	**3.121**	**0.349**	**6.637**	**0.087**	**0.010**	**0.184**
**Galliformes**	**Anseriformes**	**2.761**	**2.501**	**0.598**	**5.132**	**1410.000**	**0.996**	**10.775**	**1.000**	**21.000**	**3.764**	**0.349**	**7.335**	**0.104**	**0.010**	**0.203**
**Shorebirds**	**Nonhuman-mammal**	**2.643**	**2.531**	**0.991**	**4.629**	**277.200**	**0.979**	**21.281**	**14.000**	**30.000**	**7.434**	**4.890**	**10.479**	**0.206**	**0.136**	**0.291**
**Anseriformes**	**Raptors**	**2.628**	**2.475**	**0.984**	**4.545**	**196.286**	**0.970**	**45.234**	**26.000**	**60.000**	**15.801**	**9.082**	**20.958**	**0.438**	**0.252**	**0.581**
Galliformes	Passeriformes	2.365	2.181	0.873	4.169	95.143	0.941	7.368	1.000	16.000	2.574	0.349	5.589	0.071	0.010	0.155
**Galliformes**	**Nonhuman-mammal**	**2.267**	**2.122**	**0.492**	**4.185**	**151.333**	**0.962**	**6.759**	**1.000**	**14.000**	**2.361**	**0.349**	**4.890**	**0.065**	**0.010**	**0.136**
Raptors	Anseriformes	2.175	2.063	0.355	4.230	64.800	0.915	11.995	3.000	22.000	4.190	1.048	7.685	0.116	0.029	0.213
Anseriformes	Nonhuman-mammal	2.148	2.008	0.198	4.164	41.200	0.873	43.700	29.000	56.000	15.265	10.130	19.561	0.423	0.281	0.542
**Anseriformes**	**Shorebirds**	**2.024**	**1.879**	**0.387**	**3.799**	**102.923**	**0.945**	**33.690**	**19.000**	**47.000**	**11.768**	**6.637**	**16.417**	**0.326**	**0.184**	**0.455**
**Nonhuman - mammal**	**Galliformes**	**1.943**	**1.788**	**0.374**	**4.135**	**135.600**	**0.958**	**7.364**	**1.000**	**13.000**	**2.572**	**0.349**	**4.541**	**0.071**	**0.010**	**0.126**
Strigiformes	Raptors	1.925	1.768	0.237	4.202	26.930	0.818	9.093	1.000	19.000	3.176	0.349	6.637	0.088	0.010	0.184
**Passeriformes**	**Raptors**	**1.922**	**1.748**	**0.276**	**3.671**	**196.286**	**0.970**	**6.607**	**1.000**	**13.000**	**2.308**	**0.349**	**4.541**	**0.064**	**0.010**	**0.126**
Raptors	Shorebirds	1.843	1.761	0.021	3.381	48.462	0.890	10.215	1.000	20.000	3.568	0.349	6.986	0.099	0.010	0.194
Galliformes	Raptors	1.767	1.585	0.195	3.549	35.647	0.856	6.841	1.000	14.000	2.390	0.349	4.890	0.066	0.010	0.136
Strigiformes	Shorebirds	1.719	1.350	0.082	4.279	10.465	0.636	7.913	1.000	16.000	2.764	0.349	5.589	0.077	0.010	0.155
Shorebirds	Raptors	1.676	1.553	0.358	3.249	68.526	0.919	12.752	4.000	23.000	4.454	1.397	8.034	0.123	0.039	0.223
Strigiformes	Passeriformes	1.645	1.491	0.246	3.428	72.667	0.924	6.307	1.000	13.000	2.203	0.349	4.541	0.061	0.010	0.126
Raptors	Strigiformes	1.630	1.400	0.227	3.355	34.457	0.852	12.966	2.000	21.000	4.529	0.699	7.335	0.126	0.019	0.203
Shorebirds	Anseriformes	1.609	1.390	0.188	3.173	88.400	0.936	12.370	4.000	23.000	4.321	1.397	8.034	0.120	0.039	0.223
Nonhuman - mammal	Anseriformes	1.578	1.257	0.070	4.015	9.733	0.619	6.065	1.000	13.000	2.118	0.349	4.541	0.059	0.010	0.126
Strigiformes	Galliformes	1.569	1.155	0.032	4.223	3.077	0.339	4.346	1.000	10.000	1.518	0.349	3.493	0.042	0.010	0.097
Raptors	Nonhuman-mammal	1.563	1.217	0.043	3.509	18.414	0.754	5.693	1.000	12.000	1.989	0.349	4.192	0.055	0.010	0.116

Nonhuman - mammal	Shorebirds	1.550	1.199	0.099	3.939	6.991	0.538	4.844	1.000	11.000	1.692	0.349	3.842	0.047	0.010	0.107
**Shorebirds**	**Passeriformes**	**1.467**	**1.377**	**0.432**	**2.889**	**196.286**	**0.970**	**10.579**	**5.000**	**17.000**	**3.695**	**1.747**	**5.938**	**0.102**	**0.048**	**0.165**
Nonhuman - mammal	Raptors	1.450	1.293	0.011	3.363	20.222	0.771	7.361	1.000	14.000	2.571	0.349	4.890	0.071	0.010	0.136
Galliformes	Shorebirds	1.448	1.241	0.009	3.263	25.467	0.809	4.992	1.000	11.000	1.744	0.349	3.842	0.048	0.010	0.107
Strigiformes	Nonhuman-mammal	1.398	0.998	0.005	3.770	7.234	0.547	3.981	1.000	9.000	1.391	0.349	3.144	0.039	0.010	0.087
Raptors	Galliformes	1.393	0.950	0.014	3.926	3.316	0.356	3.613	1.000	9.000	1.262	0.349	3.144	0.035	0.010	0.087
Shorebirds	Strigiformes	1.370	1.019	0.003	4.025	9.226	0.606	5.959	1.000	12.000	2.082	0.349	4.192	0.058	0.010	0.116
Nonhuman - mammal	Strigiformes	1.350	1.024	0.023	3.472	9.391	0.610	5.693	1.000	13.000	1.989	0.349	4.541	0.055	0.010	0.126
Anseriformes	Passeriformes	1.304	0.970	0.100	3.500	7.358	0.551	24.674	11.000	35.000	8.619	3.842	12.226	0.239	0.107	0.339

Table S6: Results of BSSVS discrete trait analysis for Taxonomic host order (equal order sample 1). Table is in order of highest mean transition rate to lowest with rates having BF > 100 bolded. Rates with at least a BF > 3 are shown.

From	To	mean	median	l95hpd	h95hpd	BAYES FACTOR	POSTERIOR PROBABILITY	mean Markov Jumps	Markov Jumps 95% lhpd	Markov Jumps 95% hhpd	mean Markov Jump / tree height	Markov jumps / tree hieght lower 95%hpd	Markov jumps / tree hieght upper 95%hpd	Markov Jump / tree length	Markov Jump / tree length lower 95% hpd	Markov Jump / tree length upper 95% hpd
**Anseriformes**	**Galliformes**	**4.451**	**4.350**	**2.028**	**7.004**	**5400.000**	**0.999**	**49.279**	**30.000**	**67.000**	**15.519**	**9.448**	**21.100**	**0.523**	**0.319**	**0.712**
**Anseriformes**	**Strigiformes**	**2.987**	**2.886**	**1.293**	**5.258**	**194.222**	**0.970**	**33.088**	**15.000**	**49.000**	**10.420**	**4.724**	**15.431**	**0.351**	**0.159**	**0.520**
**Galliformes**	**Strigiformes**	**2.791**	**2.672**	**0.936**	**4.954**	**194.222**	**0.970**	**28.210**	**10.000**	**44.000**	**8.884**	**3.149**	**13.856**	**0.300**	**0.106**	**0.467**
**Galliformes**	**Nonhuman-mammal**	**2.712**	**2.620**	**1.173**	**4.723**	**409.846**	**0.986**	**26.481**	**13.000**	**37.000**	**8.339**	**4.094**	**11.652**	**0.281**	**0.138**	**0.393**
**Galliformes**	**Raptors**	**2.455**	**2.288**	**0.932**	**4.453**	**194.222**	**0.970**	**24.571**	**9.000**	**39.000**	**7.738**	**2.834**	**12.282**	**0.261**	**0.096**	**0.414**
**Nonhuman - mammal**	**Anseriformes**	**2.138**	**1.968**	**0.463**	**4.173**	**129.150**	**0.956**	**15.442**	**3.000**	**27.000**	**4.863**	**0.945**	**8.503**	**0.164**	**0.032**	**0.287**
**Galliformes**	**Passeriformes**	**2.137**	**2.046**	**0.716**	**3.505**	**239.727**	**0.976**	**20.990**	**10.000**	**30.000**	**6.610**	**3.149**	**9.448**	**0.223**	**0.106**	**0.319**
**Raptors**	**Nonhuman-mammal**	**2.106**	**2.009**	**0.333**	**3.880**	**174.200**	**0.967**	**17.918**	**5.000**	**29.000**	**5.643**	**1.575**	**9.133**	**0.190**	**0.053**	**0.308**
**Shorebirds**	**Nonhuman-mammal**	**2.001**	**1.900**	**0.740**	**3.467**	**534.600**	**0.989**	**17.302**	**9.000**	**24.000**	**5.449**	**2.834**	**7.558**	**0.184**	**0.096**	**0.255**
Galliformes	Anseriformes	1.892	1.758	0.495	3.653	96.000	0.941	17.963	4.000	30.000	5.657	1.260	9.448	0.191	0.042	0.319
**Shorebirds**	**Raptors**	**1.863**	**1.783**	**0.522**	**3.374**	**444.500**	**0.987**	**15.574**	**7.000**	**25.000**	**4.905**	**2.204**	**7.873**	**0.165**	**0.074**	**0.266**
Anseriformes	Raptors	1.855	1.739	0.383	3.679	72.348	0.923	19.340	5.000	34.000	6.091	1.575	10.707	0.205	0.053	0.361
**Raptors**	**Strigiformes**	**1.851**	**1.742**	**0.337**	**3.512**	**153.000**	**0.962**	**15.209**	**4.000**	**26.000**	**4.790**	**1.260**	**8.188**	**0.162**	**0.042**	**0.276**
Raptors	Anseriformes	1.846	1.704	0.220	3.615	69.083	0.920	15.037	2.000	26.000	4.735	0.630	8.188	0.160	0.021	0.276
**Strigiformes**	**Raptors**	**1.790**	**1.643**	**0.235**	**3.447**	**132.615**	**0.957**	**12.926**	**3.000**	**23.000**	**4.070**	**0.945**	**7.243**	**0.137**	**0.032**	**0.244**
Raptors	Shorebirds	1.688	1.566	0.260	3.486	64.208	0.915	14.188	3.000	27.000	4.468	0.945	8.503	0.151	0.032	0.287
**Strigiformes**	**Passeriformes**	**1.595**	**1.470**	**0.330**	**3.120**	**109.021**	**0.948**	**12.416**	**2.000**	**21.000**	**3.910**	**0.630**	**6.613**	**0.132**	**0.021**	**0.223**
**Anseriformes**	**Shorebirds**	**1.566**	**1.455**	**0.342**	**3.037**	**111.522**	**0.949**	**16.447**	**5.000**	**27.000**	**5.179**	**1.575**	**8.503**	**0.175**	**0.053**	**0.287**
Nonhuman - mammal	Galliformes	1.430	1.142	0.009	3.580	6.121	0.505	7.415	1.000	16.000	2.335	0.315	5.039	0.079	0.011	0.170
Passeriformes	Raptors	1.421	1.141	0.006	3.501	5.803	0.492	6.209	1.000	12.000	1.955	0.315	3.779	0.066	0.011	0.127
Strigiformes	Galliformes	1.372	1.038	0.014	3.833	3.636	0.377	7.221	1.000	17.000	2.274	0.315	5.354	0.077	0.011	0.181
Raptors	Passeriformes	1.347	1.040	0.010	3.471	7.250	0.547	8.488	1.000	17.000	2.673	0.315	5.354	0.090	0.011	0.181
Nonhuman - mammal	Raptors	1.322	1.044	0.004	3.295	5.310	0.469	6.728	1.000	15.000	2.119	0.315	4.724	0.071	0.011	0.159
Shorebirds	Strigiformes	1.320	0.793	0.003	4.003	3.086	0.340	4.692	1.000	10.000	1.478	0.315	3.149	0.050	0.011	0.106
Galliformes	Shorebirds	1.317	1.175	0.132	2.690	30.527	0.836	11.262	1.000	20.000	3.547	0.315	6.298	0.120	0.011	0.212

Strigiformes	Shorebirds	1.311	1.074	0.032	3.117	11.327	0.654	8.375	1.000	16.000	2.637	0.315	5.039	0.089	0.011	0.170
Nonhuman - mammal	Strigiformes	1.303	0.949	0.002	3.716	5.576	0.482	6.347	1.000	14.000	1.999	0.315	4.409	0.067	0.011	0.149
Nonhuman - mammal	Shorebirds	1.291	0.974	0.011	3.753	6.013	0.501	5.966	1.000	12.000	1.879	0.315	3.779	0.063	0.011	0.127
Passeriformes	Anseriformes	1.255	1.026	0.039	2.911	29.801	0.832	5.402	1.000	10.000	1.701	0.315	3.149	0.057	0.011	0.106
Raptors	Galliformes	1.240	0.988	0.002	3.038	11.495	0.657	8.312	1.000	16.000	2.618	0.315	5.039	0.088	0.011	0.170
Strigiformes	Nonhuman-mammal	1.239	0.899	0.006	3.383	6.810	0.532	6.466	1.000	14.000	2.036	0.315	4.409	0.069	0.011	0.149
**Shorebirds**	**Passeriformes**	**1.152**	**1.023**	**0.222**	**2.282**	**140.108**	**0.959**	**9.277**	**3.000**	**14.000**	**2.922**	**0.945**	**4.409**	**0.099**	**0.032**	**0.149**
Shorebirds	Anseriformes	1.118	0.873	0.002	3.217	11.439	0.656	6.597	1.000	13.000	2.077	0.315	4.094	0.070	0.011	0.138

Table S7: Results of BSSVS discrete trait analysis for Taxonomic host order (equal order sample 2). Table is in order of highest mean transition rate to lowest with rates having BF > 100 bolded. Rates with at least a BF > 3 are shown.

From	To	mean	median	l95hpd	h95hpd	BAYES FACTOR	POSTERIOR PROBABILITY	mean Markov Jumps	Markov Jumps 95% lhpd	Markov Jumps 95% hhpd	mean Markov Jump / tree height	Markov jumps / tree hieght lower 95%hpd	Markov jumps / tree hieght upper 95%hpd	Markov Jump / tree length	Markov Jump / tree length lower 95% hpd	Markov Jump / tree length upper 95% hpd
**Anseriformes**	**Galliformes**	**4.593**	**4.473**	**1.842**	**7.736**	**1075.2**	**0.994**	**48.298**	**24**	**72**	**20.316**	**10.095**	**30.286**	**0.454**	**0.225**	**0.676**
**Anseriformes**	**Strigiformes**	**3.674**	**3.455**	**1.467**	**6.572**	**210.24**	**0.972**	**39.473**	**17**	**61**	**16.604**	**7.151**	**25.659**	**0.371**	**0.16**	**0.573**
**Galliformes**	**Strigiformes**	**3.278**	**3.148**	**0.974**	**5.738**	**219.25**	**0.973**	**30.129**	**8**	**49**	**12.674**	**3.365**	**20.611**	**0.283**	**0.075**	**0.46**
**Anseriformes**	**Raptors**	**3.171**	**3.014**	**1.071**	**5.517**	**312**	**0.981**	**33.503**	**14**	**53**	**14.093**	**5.889**	**22.294**	**0.315**	**0.132**	**0.498**
**Galliformes**	**Anseriformes**	**2.974**	**2.821**	**0.685**	**5.363**	**201.923**	**0.971**	**27.024**	**6**	**48**	**11.368**	**2.524**	**20.191**	**0.254**	**0.056**	**0.451**
**Galliformes**	**Nonhuman-mammal**	**2.682**	**2.533**	**0.617**	**4.865**	**116.864**	**0.951**	**24.272**	**7**	**39**	**10.21**	**2.944**	**16.405**	**0.228**	**0.066**	**0.366**
Nonhuman - mammal	Anseriformes	2.505	2.361	0.304	4.87	64.208	0.915	17.048	3	32	7.171	1.262	13.461	0.16	0.028	0.301
Strigiformes	Raptors	2.045	1.884	0.246	4.204	48.06	0.889	14.44	3	26	6.074	1.262	10.937	0.136	0.028	0.244
**Shorebirds**	**Nonhuman-mammal**	**2.03**	**1.915**	**0.733**	**3.622**	**766.286**	**0.992**	**16.093**	**8**	**23**	**6.769**	**3.365**	**9.675**	**0.151**	**0.075**	**0.216**
**Anseriformes**	**Shorebirds**	**1.917**	**1.799**	**0.551**	**3.74**	**264.3**	**0.978**	**19.757**	**6**	**32**	**8.311**	**2.524**	**13.461**	**0.186**	**0.056**	**0.301**
Galliformes	Passeriformes	1.865	1.766	0.165	3.479	81.194	0.931	16.82	3	28	7.075	1.262	11.778	0.158	0.028	0.263
Raptors	Anseriformes	1.821	1.63	0.012	3.84	23.867	0.799	13.928	1	26	5.859	0.421	10.937	0.131	0.009	0.244
**Shorebirds**	**Raptors**	**1.74**	**1.615**	**0.329**	**3.322**	**380.143**	**0.984**	**13.668**	**4**	**22**	**5.749**	**1.683**	**9.254**	**0.128**	**0.038**	**0.207**
Anseriformes	Nonhuman-mammal	1.664	1.508	0.096	3.539	30.282	0.835	16.275	1	32	6.846	0.421	13.461	0.153	0.009	0.301
Galliformes	Shorebirds	1.65	1.488	0.215	3.383	41.009	0.872	13.486	2	24	5.673	0.841	10.095	0.127	0.019	0.225
Raptors	Shorebirds	1.63	1.499	0.172	3.494	36.905	0.86	12.477	2	22	5.248	0.841	9.254	0.117	0.019	0.207
Galliformes	Raptors	1.624	1.416	0.039	3.523	21.303	0.78	13.357	2	26	5.619	0.841	10.937	0.125	0.019	0.244
Strigiformes	Shorebirds	1.602	1.328	0.016	3.97	12.706	0.679	9.599	1	18	4.038	0.421	7.572	0.09	0.009	0.169
Raptors	Strigiformes	1.588	1.406	0.152	3.424	31.542	0.84	11.929	2	23	5.018	0.841	9.675	0.112	0.019	0.216
Nonhuman - mammal	Galliformes	1.574	1.237	0.031	4.031	9.101	0.603	8.156	1	17	3.431	0.421	7.151	0.077	0.009	0.16
Raptors	Nonhuman-mammal	1.548	1.387	0.296	3.337	77.169	0.928	11.618	2	20	4.887	0.841	8.413	0.109	0.019	0.188
Strigiformes	Passeriformes	1.535	1.425	0.183	3.086	66.08	0.917	10.874	2	19	4.574	0.841	7.992	0.102	0.019	0.178
Passeriformes	Strigiformes	1.473	1.095	0.004	4.282	3.758	0.385	4.923	1	11	2.071	0.421	4.627	0.046	0.009	0.103
Strigiformes	Galliformes	1.46	1.19	0.005	3.587	8.975	0.599	8.907	1	19	3.747	0.421	7.992	0.084	0.009	0.178
Passeriformes	Raptors	1.454	1.137	0.026	3.939	7.686	0.562	4.365	1	9	1.836	0.421	3.786	0.041	0.009	0.085
Nonhuman - mammal	Strigiformes	1.442	1.137	0.007	3.951	5.429	0.475	6.615	1	14	2.782	0.421	5.889	0.062	0.009	0.132
Nonhuman - mammal	Raptors	1.424	1.054	0.024	3.917	5.381	0.473	6.571	1	14	2.764	0.421	5.889	0.062	0.009	0.132

Anseriformes	Passeriformes	1.413	1.186	0.015	3.276	18.912	0.759	12.582	1	25	5.293	0.421	10.516	0.118	0.009	0.235
Nonhuman - mammal	Shorebirds	1.399	1.077	0.009	3.757	5.676	0.486	6.42	1	15	2.701	0.421	6.31	0.06	0.009	0.141
Strigiformes	Nonhuman-mammal	1.382	1.165	0.008	3.225	20.116	0.77	7.754	1	15	3.262	0.421	6.31	0.073	0.009	0.141
Raptors	Galliformes	1.367	1.114	0.005	3.302	10.634	0.639	8.329	1	17	3.504	0.421	7.151	0.078	0.009	0.16
Shorebirds	Strigiformes	1.227	0.806	0.004	3.752	5.881	0.495	4.89	1	10	2.057	0.421	4.206	0.046	0.009	0.094
**Shorebirds**	**Passeriformes**	**1.22**	**1.147**	**0.221**	**2.328**	**162.938**	**0.964**	**9.431**	**4**	**14**	**3.967**	**1.683**	**5.889**	**0.089**	**0.038**	**0.132**
Shorebirds	Anseriformes	1.196	0.933	0.013	2.953	20.762	0.776	7.195	1	14	3.026	0.421	5.889	0.068	0.009	0.132

Table S8: Results of BSSVS discrete trait analysis for Taxonomic host order (equal order sample 3). Table is in order of highest mean transition rate to lowest with rates having BF > 100 bolded. Rates with at least a BF > 3 are shown.

From	To	mean	median	l95hpd	h95hpd	BAYES FACTOR	POSTERIOR PROBABILITY	mean Markov Jumps	Markov Jumps 95% lhpd	Markov Jumps 95% hhpd	mean Markov Jump / tree height	Markov jumps / tree hieght lower 95%hpd	Markov jumps / tree hieght upper 95%hpd	Markov Jump / tree length	Markov Jump / tree length lower 95% hpd	Markov Jump / tree length upper 95% hpd
**Anseriformes**	**Raptors**	**5.127**	**5.039**	**2.671**	**7.870**	**6000.000**	**1.000**	**85.678**	**72.000**	**99.000**	**44.299**	**37.227**	**51.187**	**0.647**	**0.543**	**0.747**
**Anseriformes**	**Galliformes**	**4.187**	**4.061**	**2.120**	**6.373**	**6000.000**	**1.000**	**47.280**	**36.000**	**59.000**	**24.445**	**18.613**	**30.505**	**0.357**	**0.272**	**0.445**
**Raptors**	**Anseriformes**	**2.938**	**2.736**	**0.880**	**5.486**	**347.294**	**0.983**	**27.786**	**11.000**	**43.000**	**14.366**	**5.687**	**22.233**	**0.210**	**0.083**	**0.324**
Raptors	Shorebirds	1.710	1.581	0.029	3.281	42.435	0.876	15.135	7.000	23.000	7.825	3.619	11.892	0.114	0.053	0.174
**Anseriformes**	**Strigiformes**	**1.456**	**1.405**	**0.606**	**2.306**	**6000.000**	**1.000**	**31.811**	**22.000**	**40.000**	**16.448**	**11.375**	**20.681**	**0.240**	**0.166**	**0.302**
Galliformes	Anseriformes	1.302	1.035	0.002	3.313	7.903	0.568	8.462	1.000	19.000	4.375	0.517	9.824	0.064	0.008	0.143
Galliformes	Raptors	1.178	0.908	0.017	3.183	4.391	0.423	9.857	1.000	18.000	5.096	0.517	9.307	0.074	0.008	0.136
**Anseriformes**	**Shorebirds**	**1.082**	**0.989**	**0.288**	**2.083**	**456.000**	**0.987**	**20.930**	**11.000**	**29.000**	**10.822**	**5.687**	**14.994**	**0.158**	**0.083**	**0.219**
Shorebirds	Raptors	1.067	0.797	0.003	3.008	4.128	0.408	6.214	1.000	13.000	3.213	0.517	6.721	0.047	0.008	0.098
Shorebirds	Anseriformes	1.063	0.762	0.005	3.049	4.611	0.435	10.599	4.000	17.000	5.480	2.068	8.790	0.080	0.030	0.128
Raptors	Nonhuman-Mammal	1.016	0.677	0.007	3.116	3.766	0.386	7.885	2.000	13.000	4.077	1.034	6.721	0.059	0.015	0.098
Galliformes	Nonhuman-Mammal	0.983	0.765	0.003	2.571	10.410	0.634	3.114	1.000	6.000	1.610	0.517	3.102	0.024	0.008	0.045
**Anseriformes**	**Nonhuman-Mammal**	**0.710**	**0.658**	**0.183**	**1.277**	**255.130**	**0.977**	**7.070**	**1.000**	**11.000**	**3.655**	**0.517**	**5.687**	**0.053**	**0.008**	**0.083**
Raptors	Passeriformes	0.664	0.550	0.040	1.454	47.150	0.887	8.598	2.000	15.000	4.445	1.034	7.756	0.065	0.015	0.113
**Anseriformes**	**Passeriformes**	**0.569**	**0.530**	**0.138**	**1.024**	**1195.200**	**0.995**	**13.836**	**7.000**	**22.000**	**7.154**	**3.619**	**11.375**	**0.104**	**0.053**	**0.166**

Table S9: Results of BSSVS discrete trait analysis for Taxonomic host order (case proportional sample 1). Table is in order of highest mean transition rate to lowest with rates having BF > 100 bolded. Rates with at least a BF > 3 are shown.

From	To	mean	median	l95hpd	h95hpd	BAYES FACTOR	POSTERIOR PROBABILITY	mean Markov Jumps	Markov Jumps 95% lhpd	Markov Jumps 95% hhpd	mean Markov Jump / tree height	Markov jumps / tree hieght lower 95%hpd	Markov jumps / tree hieght upper 95%hpd	Markov Jump / tree length	Markov Jump / tree length lower 95% hpd	Markov Jump / tree length upper 95% hpd
**Anseriformes**	**Raptors**	**6.506**	**6.299**	**3.435**	**9.973**	**6000.000**	**1.000**	**87.856**	**75.000**	**99.000**	**34.776**	**29.687**	**39.187**	**0.603**	**0.515**	**0.680**
**Anseriformes**	**Galliformes**	**4.443**	**4.280**	**2.520**	**7.024**	**6000.000**	**1.000**	**42.613**	**30.000**	**52.000**	**16.867**	**11.875**	**20.583**	**0.293**	**0.206**	**0.357**
**Raptors**	**Anseriformes**	**3.009**	**2.830**	**0.986**	**5.906**	**208.500**	**0.972**	**34.512**	**14.000**	**54.000**	**13.661**	**5.542**	**21.375**	**0.237**	**0.096**	**0.371**
**Anseriformes**	**Shorebirds**	**2.133**	**2.023**	**0.931**	**3.447**	**6000.000**	**1.000**	**29.608**	**14.000**	**43.000**	**11.720**	**5.542**	**17.020**	**0.203**	**0.096**	**0.295**
Galliformes	Anseriformes	1.696	1.451	0.050	4.017	16.664	0.735	7.348	1.000	17.000	2.908	0.396	6.729	0.050	0.007	0.117
**Anseriformes**	**Strigiformes**	**1.528**	**1.437**	**0.533**	**2.554**	**6000.000**	**0.999**	**27.804**	**17.000**	**38.000**	**11.006**	**6.729**	**15.041**	**0.191**	**0.117**	**0.261**
Galliformes	Raptors	1.358	1.036	0.035	3.744	3.734	0.384	6.912	1.000	13.000	2.736	0.396	5.146	0.047	0.007	0.089
Shorebirds	Anseriformes	1.357	1.048	0.005	3.559	5.708	0.488	8.537	1.000	18.000	3.379	0.396	7.125	0.059	0.007	0.124
Raptors	Strigiformes	1.355	1.173	0.114	3.012	32.500	0.844	17.417	6.000	28.000	6.894	2.375	11.083	0.120	0.041	0.192
Raptors	Nonhuman-Mammal	1.329	0.942	0.003	3.853	3.032	0.336	4.321	1.000	10.000	1.710	0.396	3.958	0.030	0.007	0.069
Raptors	Shorebirds	1.278	0.990	0.006	3.764	3.326	0.357	21.252	7.000	36.000	8.412	2.771	14.250	0.146	0.048	0.247
Galliformes	Nonhuman-Mammal	1.237	0.922	0.007	3.621	3.059	0.338	2.925	1.000	6.000	1.158	0.396	2.375	0.020	0.007	0.041
Strigiformes	Raptors	1.203	0.862	0.001	3.389	4.145	0.409	12.006	1.000	22.000	4.752	0.396	8.708	0.082	0.007	0.151
Shorebirds	Nonhuman-Mammal	1.140	0.872	0.003	2.965	6.307	0.512	3.665	1.000	6.000	1.451	0.396	2.375	0.025	0.007	0.041
Passeriformes	Galliformes	1.118	0.793	0.003	3.318	5.440	0.476	3.573	1.000	7.000	1.414	0.396	2.771	0.025	0.007	0.048
**Anseriformes**	**Nonhuman-Mammal**	**0.995**	**0.921**	**0.198**	**1.709**	**181.688**	**0.968**	**11.096**	**6.000**	**15.000**	**4.392**	**2.375**	**5.937**	**0.076**	**0.041**	**0.103**
**Anseriformes**	**Passeriformes**	**0.740**	**0.694**	**0.220**	**1.367**	**394.400**	**0.985**	**15.569**	**8.000**	**23.000**	**6.163**	**3.167**	**9.104**	**0.107**	**0.055**	**0.158**

Table S10: Results of BSSVS discrete trait analysis for Taxonomic host order (case proportional sample 2). Table is in order of highest mean transition rate to lowest with rates having BF > 100 bolded. Rates with at least a BF > 3 are shown.

From	To	mean	median	l95hpd	h95hpd	BAYES FACTOR	POSTERIOR PROBABILITY	mean Markov Jumps	Markov Jumps 95% lhpd	Markov Jumps 95% hhpd	mean Markov Jump / tree height	Markov jumps / tree hieght lower 95%hpd	Markov jumps / tree hieght upper 95%hpd	Markov Jump / tree length	Markov Jump / tree length lower 95% hpd	Markov Jump / tree length upper 95% hpd
**Anseriformes**	**Raptors**	**6.016**	**5.933**	**3.202**	**9.178**	**6000.000**	**1.000**	**83.491**	**69.000**	**99.000**	**36.763**	**30.382**	**43.592**	**0.612**	**0.506**	**0.726**
**Anseriformes**	**Galliformes**	**4.648**	**4.525**	**2.393**	**7.064**	**6000.000**	**1.000**	**53.319**	**42.000**	**61.000**	**23.478**	**18.494**	**26.860**	**0.391**	**0.308**	**0.447**
**Raptors**	**Anseriformes**	**2.904**	**2.684**	**0.915**	**5.356**	**423.000**	**0.986**	**16.650**	**3.000**	**31.000**	**7.331**	**1.321**	**13.650**	**0.122**	**0.022**	**0.227**
**Anseriformes**	**Shorebirds**	**1.724**	**1.632**	**0.566**	**2.983**	**6000.000**	**1.000**	**25.697**	**13.000**	**37.000**	**11.315**	**5.724**	**16.292**	**0.188**	**0.095**	**0.271**
**Anseriformes**	**Strigiformes**	**1.540**	**1.472**	**0.659**	**2.660**	**6000.000**	**1.000**	**21.595**	**12.000**	**29.000**	**9.509**	**5.284**	**12.769**	**0.158**	**0.088**	**0.213**
Raptors	Shorebirds	1.376	1.197	0.084	3.069	15.761	0.724	18.455	8.000	28.000	8.126	3.523	12.329	0.135	0.059	0.205
Shorebirds	Anseriformes	1.181	0.988	0.043	2.996	64.659	0.915	15.695	5.000	25.000	6.911	2.202	11.008	0.115	0.037	0.183
**Raptors**	**Strigiformes**	**1.155**	**1.012**	**0.053**	**2.409**	**116.571**	**0.951**	**20.145**	**11.000**	**29.000**	**8.870**	**4.844**	**12.769**	**0.148**	**0.081**	**0.213**
**Anseriformes**	**Nonhuman-Mammal**	**1.029**	**0.986**	**0.413**	**1.852**	**6000.000**	**1.000**	**18.171**	**9.000**	**27.000**	**8.001**	**3.963**	**11.889**	**0.133**	**0.066**	**0.198**
Passeriformes	Raptors	0.961	0.720	0.014	2.696	11.014	0.647	4.772	1.000	10.000	2.101	0.440	4.403	0.035	0.007	0.073
**Anseriformes**	**Passeriformes**	**0.731**	**0.696**	**0.186**	**1.341**	**1195.200**	**0.995**	**12.717**	**5.000**	**20.000**	**5.599**	**2.202**	**8.806**	**0.093**	**0.037**	**0.147**

Table S11: Results of BSSVS discrete trait analysis for Taxonomic host order (case proportional sample 3). Table is in order of highest mean transition rate to lowest with rates having BF > 100 bolded. Rates with at least a BF > 3 are shown.

Analysis	ratio	Transitions to Domestic	Transitions to Wild
2-state	1:1	46	40
2-state	1:1.5	66	29
2-state	1:2	79	16
2-state	1:2.5	99	4
2-state	1:3	106	4
2-state with turkey	1:1	61	51
2-state with turkey	1:1.5	109	26
2-state with turkey	1:2	113	4

Table S12: Transitions into Domestic and Wild for each ratio dataset for two state rarefaction and two state rarefaction with turkey sequences.

From	To	Transition count
Wild	Domestic	48
Wild	Turkey	42
Domestic	Wild	5
Domestic	Turkey	18
Turkey	Domestic	38
Turkey	Wild	1

Table S13: Number of transitions between states in three state rarefaction of wild, domestic, and turkey for the 1:2 domestic:wild with turkey sequence dataset.

From	To	mean	median	l95hpd	h95hpd	BAYES_FACTOR	POSTERIOR PROBABILITY
**Wild**	**Backyard_bird**	**1.86774162**	**1.70511383**	**0.37454847**	**3.68662003**	**18000**	**1**
**Wild**	**Commercial**	**1.46084104**	**1.31711214**	**0.26387335**	**3.00326823**	**18000**	**1**
Backyard_bird	Commercial	0.95115148	0.81135468	0.07030257	2.20310188	87.5621891	0.97766915
Backyard_bird	Wild	0.59728953	0.41999835	0.00047605	1.71248402	7.84792123	0.79691145
Commercial	Backyard_bird	0.58676046	0.29058374	3.52E-05	2.18585542	2.50726089	0.55627153
Commercial	Wild	0.51687069	0.26402531	0.00010671	1.99546369	3.66278704	0.64681702

Table S14. Discrete trait rates for backyard bird wild bird titration analysis (25% of all wild bird sequence). Table is in order of highest mean transition rate to lowest with rates having BF > 100 bolded. Rates with at least a BF > 3 are shown.

From	To	mean	median	l95hpd	h95hpd	BAYES_FACTOR	POSTERIOR PROBABILITY
**Wild**	**Backyard_bird**	**1.91959767**	**1.75610576**	**0.39212384**	**3.81857375**	**18000**	**1**
**Wild**	**Commercial**	**1.71435411**	**1.5565693**	**0.3141297**	**3.48816005**	**18000**	**1**
Backyard_bird	Commercial	0.72271333	0.56016402	0.00049205	1.90186488	12.5529507	0.86257083
Commercial	Wild	0.57838557	0.25387721	1.88E-05	2.25248367	1.92799476	0.49083435
Backyard_bird	Wild	0.56892638	0.36543149	0.00035904	1.81943486	5.7561396	0.74213976
Commercial	Backyard_bird	0.49228375	0.24128113	1.86E-05	1.8770157	3.86575432	0.65903788

Table S15 Discrete trait rates for backyard bird wild bird titration analysis (50% of all wild bird sequence). Table is in order of highest mean transition rate to lowest with rates having BF > 100 bolded. Rates with at least a BF > 3 are shown.

From	To	mean	median	l95hpd	h95hpd	BAYES_FACTOR	POSTERIOR PROBABILITY
**Wild**	**Backyard_bird**	**1.84672556**	**1.68062813**	**0.2989798**	**3.81025501**	**18000**	**1**
**Wild**	**Commercial**	**1.58010327**	**1.43058414**	**0.24177847**	**3.28214739**	**18000**	**1**
Backyard_bird	Wild	0.81861356	0.50058483	1.08E-05	2.66432008	0.72964367	0.26730363
Backyard_bird	Commercial	0.64099347	0.50335486	0.0056189	1.63180822	18.9569267	0.90456616
Commercial	Wild	0.60637747	0.24737791	1.03E-06	2.40045423	1.71865317	0.46217087
Commercial	Backyard_bird	0.48361884	0.20683227	9.64E-06	1.94383423	3.45680509	0.63348517

Table S16. Discrete trait rates for backyard bird wild bird titration analysis (75% of all wild bird sequence). Table is in order of highest mean transition rate to lowest with rates having BF > 100 bolded. Rates with at least a BF > 3 are shown.

From	To	mean	median	l95hpd	h95hpd	BAYES FACTOR	POSTERIOR PROBABILITY	mean Markov Jumps	Markov Jumps 95% lhpd	Markov Jumps 95% hhpd	mean Markov Jump / tree height	Markov jumps / tree hieght lower 95%hpd	Markov jumps / tree hieght upper 95%hpd	Markov Jump / tree length	Markov Jump / tree length lower 95% hpd	Markov Jump / tree length upper 95% hpd
**Wild**	**Backyard_bird**	**1.825**	**1.644**	**0.283**	**3.787**	**18000.000**	**1.000**	**42.669**	**35.000**	**49.000**	**48.963**	**40.163**	**56.228**	**0.755**	**0.619**	**0.867**
**Wild**	**Commercial**	**1.601**	**1.441**	**0.233**	**3.356**	**18000.000**	**1.000**	**40.506**	**35.000**	**46.000**	**46.481**	**40.163**	**52.785**	**0.716**	**0.619**	**0.814**
Backyard_bird	Wild	0.859	0.545	3.260E-05	2.727	0.424	0.175	1.748	1.000	4.000	2.006	1.148	4.590	0.031	0.018	0.071
Backyard_bird	Commercial	0.639	0.405	3.090E-06	2.053	4.584	0.696	2.334	1.000	4.000	2.678	1.148	4.590	0.041	0.018	0.071
Commercial	Backyard_bird	0.563	0.229	1.880E-06	2.317	2.137	0.517	1.257	1.000	2.000	1.442	1.148	2.295	0.022	0.018	0.035
Commercial	Wild	0.519	0.209	1.090E-05	2.072	2.649	0.570	1.262	1.000	2.000	1.448	1.148	2.295	0.022	0.018	0.035

Table S17 Discrete trait rates for backyard bird wild bird titration analysis (100% of all wild bird sequence). Table is in order of highest mean transition rate to lowest with rates having BF > 100 bolded. Rates with at least a BF > 3 are shown.

Table S20. Acknowledgments table for GISAID isolates used in these analyses. Please visit GitHub repository for acknowledgements table.
