# Supplementary material for: Ecology and spread of the North American H5N1 epizootic
Source: Nature. 2025 Nov 12;649(8096):432–41. doi: 10.1038/s41586-025-09737-x (PMC12779553; doi:10.1038/s41586-025-09737-x)
Supplement: Supplementary file 2 — Reporting Summary [file 41586_2025_9737_MOESM2_ESM.pdf]

nature portfolio

Corresponding author(s): Louise H. Moncla

Last updated by author(s): Jul 18, 2025

# Reporting Summary

Nature Portfolio wishes to improve the reproducibility of the work that we publish. This form provides structure for consistency and transparency in reporting. For further information on Nature Portfolio policies, see our Editorial Policies and the Editorial Policy Checklist.

## Statistics

For all statistical analyses, confirm that the following items are present in the figure legend, table legend, main text, or Methods section.

| n/a | Confirmed | |
|---|---|---|
| ☐ | ☒ | The exact sample size (*n*) for each experimental group/condition, given as a discrete number and unit of measurement |
| ☒ | ☐ | A statement on whether measurements were taken from distinct samples or whether the same sample was measured repeatedly |
| ☐ | ☒ | The statistical test(s) used AND whether they are one- or two-sided<br>*Only common tests should be described solely by name; describe more complex techniques in the Methods section.* |
| ☐ | ☒ | A description of all covariates tested |
| ☐ | ☒ | A description of any assumptions or corrections, such as tests of normality and adjustment for multiple comparisons |
| ☐ | ☒ | A full description of the statistical parameters including central tendency (e.g. means) or other basic estimates (e.g. regression coefficient) AND variation (e.g. standard deviation) or associated estimates of uncertainty (e.g. confidence intervals) |
| ☐ | ☒ | For null hypothesis testing, the test statistic (e.g. *F*, *t*, *r*) with confidence intervals, effect sizes, degrees of freedom and *P* value noted<br>*Give P values as exact values whenever suitable.* |
| ☐ | ☒ | For Bayesian analysis, information on the choice of priors and Markov chain Monte Carlo settings |
| ☒ | ☐ | For hierarchical and complex designs, identification of the appropriate level for tests and full reporting of outcomes |
| ☐ | ☒ | Estimates of effect sizes (e.g. Cohen's *d*, Pearson's *r*), indicating how they were calculated |

*Our web collection on statistics for biologists contains articles on many of the points above.*

## Software and code

Policy information about availability of computer code

| | |
|---|---|
| Data collection | We downloaded all available nucleotide sequence data and associated meta-data for the Hemagglutinin protein of all HPAI clade 2.3.4.4b H5Nx viruses from the GISAID database on 2023-11-25. We downloaded the AVONET database for avian ecology data and merged it to available host metadata from GISAID for each sequence. We used the species if provided to match the species indicated in the AVONET database. If host metadata in GISAID was defined using common name for a bird, we determined the taxonomic species name and used that for further merging with the AVONET data. Data for detections of HPAI in North America were collected from USDA APHIS. Reports for mammals, wild birds, and domestic poultry were all downloaded (download date: 2023-11-25). |
| Data analysis | For each subset of the data described for further phylodynamic modeling the following process was followed. We first aligned sequences using MAFFT v7.5, sequence alignments were visually inspected using Geneious and sequences causing significant gaps were removed and nucleotides before the start codon and after the stop codon were removed. We de-duplicated identical sequences collected on the same day (retaining identical sequences that occurred on different days). We identified and removed temporal outliers for all genomic datasets by performing initial phylogenetic reconstruction in a maximum likelihood framework using IQtree v.1.6.12 and used the program TimeTree v 0.11.2 was used to remove temporal outliers. Bayesian phylogenetic reconstructions and analyses were performed using BEAST v.1.10.4. We performed Bayesian phylogenetic reconstruction for each dataset prior to discrete trait diffusion modeling to estimate a posterior set of empirical trees. The following priors and settings were used for each subset of the sequence data. We used the HKY nucleotide substitution model with gamma-distributed rate variation among sites and lognormal relaxed molecular clock model. The Bayesian SkyGrid coalescent was used with the number of grid points corresponding to the number of weeks between the earliest and latest collected sample (e.g for a dataset collected between 2021-11-04 and 2023-08-11 we would set 92 grid points). We initially ran four independent MCMC chains with a chain-length of 100 million states logging every 10000 states. We diagnosed the combined results of the independent runs diagnosed Tracer v1.7.2. to ensure adequate ESS (ESS > 200) and reasonable estimates for parameters. If ESS was inadequate additional independent MCMC runs were run increasing chain length to 150 million states, sampling every 15000 states were performed. We combined the tree files from each |

independent MCMC run removing 10-30% burn-in and resampling to get a tree file with between 9000 and 10000 posterior trees using Logcombiner v1.10.4. A posterior sample of 500 trees was extracted and used as empirical tree sets in discrete trait diffusion modeling. We defined discrete traits for use in discrete trait diffusion modeling based on the available sequence metadata and merged AVONET data. For each discrete trait dataset, we used an asymmetric continuous time Markov chain discrete trait diffusion model and implemented the Bayesian stochastic search variable selection (BSSVS) to determine the most parsimonious diffusion network. We calculate the transition rate as a realization of the CTMC process by dividing the number of markov jumps by the tree height (branch length from the earliest tip to the root of the tree) , and separately, by tree length (sum of all branch lengths). For each pairwise transition rate, we calculate the level of Bayes Factor (BF) support that the given rate has.

For manuscripts utilizing custom algorithms or software that are central to the research but not yet described in published literature, software must be made available to editors and reviewers. We strongly encourage code deposition in a community repository (e.g. GitHub). See the Nature Portfolio guidelines for submitting code & software for further information.

## Data

Policy information about availability of data

All manuscripts must include a data availability statement. This statement should provide the following information, where applicable:

- Accession codes, unique identifiers, or web links for publicly available datasets
- A description of any restrictions on data availability
- For clinical datasets or third party data, please ensure that the statement adheres to our policy

All analytical scripts, metadata annotations, and BEAST XMLs used in this analysis can be found at the following GitHub repository: https://github.com/moncla-lab/North-American-HPAI

All data that was used in this analysis were sourced from public databases. Acknowledgement table for GISAID isolates used in this analysis can be found in Table S20.

Several of the analyses presented have also been publicly made available using a maximum likelihood framework through the Nextstrain pipeline and a narrative of this work can be found in the following link: https://nextstrain.org/community/narratives/moncla-lab/nextstrain-narrative-hpai-north-america@main/HPAI-in-North-America

## Research involving human participants, their data, or biological material

Policy information about studies with human participants or human data. See also policy information about sex, gender (identity/presentation), and sexual orientation and race, ethnicity and racism.

| | |
|---|---|
| Reporting on sex and gender | N/A |
| Reporting on race, ethnicity, or other socially relevant groupings | N/A |
| Population characteristics | N/A |
| Recruitment | N/A |
| Ethics oversight | N/A |

Note that full information on the approval of the study protocol must also be provided in the manuscript.

# Field-specific reporting

Please select the one below that is the best fit for your research. If you are not sure, read the appropriate sections before making your selection.

☒ Life sciences ☐ Behavioural & social sciences ☐ Ecological, evolutionary & environmental sciences

For a reference copy of the document with all sections, see nature.com/documents/nr-reporting-summary-flat.pdf

# Life sciences study design

All studies must disclose on these points even when the disclosure is negative.

| | |
|---|---|
| Sample size | We downloaded all available nucleotide sequence data and associated meta-data for the Hemagglutinin protein of all HPAI clade 2.3.4.4b H5Nx viruses from the GISAID database on 2023-11-2589. For each subset of the data described for further phylodynamic modeling the following process was followed. We first aligned sequences using MAFFT v7.5.20, sequence alignments were visually inspected using Geneious and sequences causing significant gaps were removed and nucleotides before the start codon and after the stop codon were removed. We de-duplicated identical sequences collected on the same day (retaining identical sequences that occurred on different days). We identified and removed temporal outliers for all genomic datasets by performing initial phylogenetic reconstruction in a maximum |

likelihood framework using IQtree v.1.6.12 and used the program TimeTree v 0.11.2 was used to remove temporal outliers and to assess the clockliness of the dataset prior to Bayesian phylogenetic reconstruction. This resulted in a dataset of 1824 sequences that were used in further analyses (Figure S24).

| | |
|---|---|
| Data exclusions | Temporal outliers determined by initial phylogenetic analysis were removed. We de-duplicated identical sequences collected on the same day (retaining identical sequences that occurred on different days). We identified and removed temporal outliers for all genomic datasets by performing initial phylogenetic reconstruction in a maximum likelihood framework using IQtree v.1.6.12 and used the program TimeTree v 0.11.2 was used to remove temporal outliers and to assess the clockliness of the dataset prior to Bayesian phylogenetic reconstruction Data for discrete trait diffusion models were subsampled based on availability of data for given discrete traits. |
| Replication | We performed at least three independent runs of analyses using the models and parameters described in the empirical tree analysis section above. All titration replicates were performed using an MCMC chain length of 100 million states sampling every 10,000 states. |
| Randomization | Random sub-sampling of data was performed for discrete trait datasets as well as random case proportional subsamplig for analyses of host order transmission. |
| Blinding | N/A |

# Reporting for specific materials, systems and methods

We require information from authors about some types of materials, experimental systems and methods used in many studies. Here, indicate whether each material, system or method listed is relevant to your study. If you are not sure if a list item applies to your research, read the appropriate section before selecting a response.

## Materials & experimental systems

| n/a | Involved in the study | |
|---|---|---|
| ☒ | ☐ | Antibodies |
| ☒ | ☐ | Eukaryotic cell lines |
| ☒ | ☐ | Palaeontology and archaeology |
| ☒ | ☐ | Animals and other organisms |
| ☒ | ☐ | Clinical data |
| ☒ | ☐ | Dual use research of concern |
| ☒ | ☐ | Plants |

## Methods

| n/a | Involved in the study | |
|---|---|---|
| ☒ | ☐ | ChIP-seq |
| ☒ | ☐ | Flow cytometry |
| ☒ | ☐ | MRI-based neuroimaging |

## Plants

| | |
|---|---|
| Seed stocks | N/A |
| Novel plant genotypes | N/A |
| Authentication | N/A |
